# Annotated features of domestic cat – *Felis catus* genome

**DOI:** 10.1186/2047-217X-3-13

**Published:** 2014-08-05

**Authors:** Gaik Tamazian, Serguei Simonov, Pavel Dobrynin, Alexey Makunin, Anton Logachev, Aleksey Komissarov, Andrey Shevchenko, Vladimir Brukhin, Nikolay Cherkasov, Anton Svitin, Klaus-Peter Koepfli, Joan Pontius, Carlos A Driscoll, Kevin Blackistone, Cristina Barr, David Goldman, Agostinho Antunes, Javier Quilez, Belen Lorente-Galdos, Can Alkan, Tomas Marques-Bonet, Marylin Menotti-Raymond, Victor A David, Kristina Narfström, Stephen J O’Brien

**Affiliations:** 1 Theodosius Dobzhansky Center for Genome Bioinformatics, St. Petersburg State University, 199004, St. Petersburg, Russia; 2 Laboratory of Neurogenetics, NIAAA, 5625 Fishers Lane, 20852 Rockville, MD, USA; 3 CIIMAR — Interdisciplinary Centre of Marine and Environmental Research, University of Porto, Rua dos Bragas, n. 289, 4050–123 Porto, Portugal; 4 Department of Animal and Food Science, Veterinary Molecular Genetics Service, Universitat Autónoma de Barcelona, 08003 Barcelona, Catalonia, Spain; 5 IBE, Institute of Evolutionary Biology, Universitat Pompeu Fabra-CSIC, PRBB (The Barcelona Biomedical Research Park), 08003 Barcelona, Catalonia, Spain; 6 Department of Computer Engineering, Bilkent University, 06800 Ankara, Turkey; 7 Laboratory of Genomic Diversity, Frederick National Laboratory for Cancer Research, 21702 Frederick, MD, USA; 8 Department of Veterinary Medicine and Surgery, College of Veterinary Medicine, University of Missouri, 08028 Columbia, MO, USA; 9 Oceanographic Center, Nova Southeastern University, 33004 Ft Lauderdale, FL, USA

**Keywords:** *Felis catus*, Domestic cat, *Felis silvestris silvestris*, European wildcat, Genome sequence, Annotation, Assembly

## Abstract

**Background:**

Domestic cats enjoy an extensive veterinary medical surveillance which has described nearly 250 genetic diseases analogous to human disorders. Feline infectious agents offer powerful natural models of deadly human diseases, which include feline immunodeficiency virus, feline sarcoma virus and feline leukemia virus. A rich veterinary literature of feline disease pathogenesis and the demonstration of a highly conserved ancestral mammal genome organization make the cat genome annotation a highly informative resource that facilitates multifaceted research endeavors.

**Findings:**

Here we report a preliminary annotation of the whole genome sequence of Cinnamon, a domestic cat living in Columbia (MO, USA), bisulfite sequencing of Boris, a male cat from St. Petersburg (Russia), and light 30× sequencing of Sylvester, a European wildcat progenitor of cat domestication. The annotation includes 21,865 protein-coding genes identified by a comparative approach, 217 loci of endogenous retrovirus-like elements, repetitive elements which comprise about 55.7*%* of the whole genome, 99,494 new SNVs, 8,355 new indels, 743,326 evolutionary constrained elements, and 3,182 microRNA homologues. The methylation sites study shows that 10.5*%* of cat genome cytosines are methylated. An assisted assembly of a European wildcat, *Felis silvestris silvestris*, was performed; variants between *F. silvestris* and *F. catus* genomes were derived and compared to *F. catus*.

**Conclusions:**

The presented genome annotation extends beyond earlier ones by closing gaps of sequence that were unavoidable with previous low-coverage shotgun genome sequencing. The assembly and its annotation offer an important resource for connecting the rich veterinary and natural history of cats to genome discovery.

## Data description

The genome of a female Abyssinian cat (“Cinnamon” who resides at the University of Missouri-Columbia, USA) was sequenced at 1.8 × and 3.0 × whole genome shotgun (WGS) coverage at Agencourt Inc. Fca-6.2, an additional 12 × coverage of 454 reads and BAC ends was sequenced, assembled with CABOG [1] and analysed at Washington University, St. Louis (USA) [2]. Fca-6.2 is anchored to chromosome coordinates with two physical framework maps, a radiation hybrid map [3] and a short tandem repeat (STR) linkage map [4]. Further, 1943 distinct sites identified in a recently built linkage map using a single nucleotide polymorphism (SNP) genotyping array including ≈60,000 SNPs from an Illumina custom cat genotyping array are also mapped to the assembly.

Here we present a genome browser, Genome Annotation Resource Fields — GARfield [5], which displays the Fca-6.2 assembly and included annotated genome features. In Table 1 we list the features of GARfield annotated in the cat genome assembly which are described and illustrated in the Additional file 1 of this Data Note. The genome features detected in Fca-6.2 include a merged list of 21,865 genes derived from a comparative gene identification strategy using BLAST alignments between gene exons of reference genome from eight reference mammalian gene maps (human, chimpanzee, macaque, dog, cow, horse, rat, and mouse) obtained from the Ensembl Gene 75 database [6]. In addition, the whole genome methylation sites and a methylome bisulfite sequence pattern of cat whole blood cells is presented, previewing epigenetic profiling in important complex disease associations, including diseases with viral and neoplastic etiology.

**Table 1:** Annotated cat genome features available as genome browser tracks for GARfield and UCSC genome browsers

**Feature**	**Additional file****1**
I. Assembly of *Felis catus* genome Fca-6.2	
II. Gene annotation	Tables S1–S7
III. Domestic cat DNA variants	Tables S8, S9; Figures S2, S3
IV. Repeats content	Tables S10–S16; Figures S4–S13
V. Nuclear mitochondrial (Numt) pseudo gene fragments	Figure S14
VI. Evolutionary constrainedelements (ECE)	Tables S17, S18
VII. Feline endogenous retrovirus-like elements	Table S19; Figure S18
VIII. Methylation sites	Table S20
IX. MicroRNA	Table S21
X. Variants between *F. silvestris* and *F. catus*.	

Approximately 55.7*%* of the cat genome is composed of repetitive elements of familiar classes (LINEs, SINEs, satellite DNA, LTRs and others). We report more than 25 novel families of complex tandem repeat elements in the cat genome uncovered by multiple repeat detection algorithms. We searched for STR-microsatellite loci useful in population and forensic applications. Putative PCR primers for 53,710 STR loci are annotated. We also mapped known feline endogenous retroviral loci (full length RD114, FeLV, FERV) and detected 125 kb of partial retroviral genome sequences dispersed across the cat genome. Nuclear mitochondrial (Numt) DNA pseudogenes derived from ancient transposition from cytoplasmic mitochondrial chromosomes to nuclear chromosomal positions comprise 176 kb in addition to the Lopez-Numt, a 7.8 kb element tandem-repeated 38–76 times on Chromosome D2 previously described in the 1.8× analysis of Cinnamon’s genome [7].

The earlier 3,078,438 feline single nucleotide variants (SNVs) [7,8] from largely non-repetitive regions of the cat genome are supplemented with a new group of 99,494 newly annotated SNPs plus 8,355 detected indels. In addition, we performed an assisted assembly with a 40× Illumina SOLID DNA sequence coverage of Sylvester, a European wildcat, *F. silvestris silvestris*, a wild representative of the species from which cats were domesticated approximately 10,000 years ago [9]. Genome variations (SNVs and indels) between *F. silvestris* and *F. catus* SNPs are reported here and both species’ genomes and their associated data have been uploaded to the GARfield genome browser (see Availability of supporting data section).

Our annotation resolved cat homologues of 743,362 evolutionarily constrained elements (ECEs) recently identified in the human genome by alignment to 29 different mammalian genomes [10] and these were compared to the conserved sequence blocks obtained by the reciprocal best match (RBM) screen for cat genes with seven mammalian genomes (human, chimp, macaque, dog, cow, rat and mouse). A conservative alignment approach implicated 54*%* of the human ECE sequence comprising ≈3*%* of the cat genome. A total of 3,182 feline microRNA (miRNA) homologues were detected and mapped based upon homology to miRNA sequences from 36 species with miRNA sequence described in the miRBase database [11]. Finally we screened the genome sequence for copy number variation and segmental duplications. All annotated features listed in Table 1 are described in detail in Additional file 1 and tracked in the GARfield genome browser.

## Availability of supporting data

The assembly sequences are available at NCBI RefSeq database (accession numbers #PRJNA175699 and #PRJNA253950). The annotated features are available in the Genome Association Resource Fields (GARfield) genome browser http://garfield.dobzhanskycenter.org and the UCSC Genome Browser (http://genome.ucsc.edu), which links to a Dobzhansky Center Hub (http://public.dobzhanskycenter.ru/Hub/hub.txt) (See Section 2 of Additional file 1 for instructions). Supplementary tables and figures that refer to GARfield features are given in Additional file 1 and listed in Table 1.

Sequence and variation data is available in NCBI (SAMN02795853 for Boris the cat and SAMN02898152 for wildcat) and supporting data is also available in the GigaDB repository [12].

## Abbreviations

ECE: Evolutionary constrained element; SNP: Single nucleotide polymorphism; SNV: Single nucleotide variant; STR: Short tandem repeat.

## Competing interests

The authors declare that they have no competing interests.

## Authors’ contributions

SJO developed the overall project design. CAD, KB, CB and DG provided the sequence and analyses of *F. silvestris*. MMR and VAD provided the tissue and cat selection tissue for transcriptome and genome variation discovery. KN provided the tissues and sample for Cinnamon, the individual cat whose genome was assembled. SJO, AS and GT wrote the paper. GT performed gene annotation. SS designed the GARfield genome browser. GT prepared tracks of the annotated genome features. PD, AA and GT described DNA variants (SNVs and indels). AL, AK and GT annotated repetitive elements. JP designed STR primers. AM and GT detected evolutionary constrained elements. AS, AS, KPK and GT annotated endogenous retrovirus-like elements. VB, GT and NC analyzed genome methylation. PD and GT detected microRNA homologues. PD and AA searched for Numts. TMB, CA, BLG, and JQ provided the sequence and analyses of copy number variation for *F. catus*. PD assembled *F. silvestris* genome, derived SNVs from it and compared them to *F. catus* SNVs. All authors read and approved the final manuscript.

## Supplementary Material

Additional file 1 Supplementary materials. Click here for file
