# Supplementary material for: Annotated features of domestic cat – Felis catus genome
Source: Gigascience. 2014 Aug 5;3:13. doi: 10.1186/2047-217X-3-13 (PMC4138527; doi:10.1186/2047-217X-3-13)

April 24, 2013

Supplemental Methods and Materials

**I. Assemblies of the *Felis catus* genome**

The genome of a female Abyssinian cat (“Cinnamon” who resides at the University of Missouri-Columbia) was sequenced at $1.8\times$ and $3.0\times$ whole genome shotgun (WGS) coverage at Agencourt Inc. Initially a total of 8,027,672 sequence reads (84% from plasmids and 16% from fosmid paired ends) were assembled to 817,956 contigs (N50=2.4kb) and 217,790 scaffolds ( N50=117kb) with PHUSION and ARACHNE ( 1). To fill in widespread homozygous segments in Cinnamon’s genome derived from a history of inbreeding for SNP discovery, six additional domestic cats and one wildcat (*Felis silvestris*) were sequenced at Agencourt and combined with Cinnamon to produce 2.8-fold coverage genome with increased size for contigs (N50=4.6kb) and scaffolds (N50=162kb) and 3 million discovered SNPs (2). In 2011, Fca-6.2, an additional 12x coverage of 454 reads and BAC ends was sequenced, assembled with CABOG and analyzed at Washington University, St. Louis (3,4); (Montague M. et al submitted). Fca-6.2 is anchored to chromosome coordinates with two physical framework maps, a radiation hybrid map (5) and a STR linkage map (6). Further, 1,952 distinct sites identified in a recently built linkage map using a SNP genotyping array including ~60,000 SNPs from an Illumina custom cat genotyping array are also mapped to the assembly (Makunin A. et al in prep.; Li G. et al in prep.).

**II. GARfield Genome Browser for domestic cat genome Fca-6.2**

Annotated features for a domestic cat genome Fca-6.2 assembly have been deposited in interactive web-based Genome Annotation Resource Fields 2 (GARfield browser - [http://GARfield.dobzhanskycenter.org](http://garfield2.dobzhanskycenter.org)) at the Theodosius Dobzhansky Center for Genome Bioinformatics, St. Petersburg State University. The GARfield browser is a JBrowse extension of [GARFIELD browser - http://lgd.abcc.ncifcrf.gov/cgi-bin/gbrowse/cat/](file:///C:\Downloads\GARFIELD%20browser%20%20-%20http:\lgd.abcc.ncifcrf.gov\cgi-bin\gbrowse\cat\) (7,8) based on AJAX technology and implemented in BioPerl language combined with JavaScript. GARfield can be installed on Apache 2-based web server with preinstalled Perl 5.8 and above. JBrowse is faster and more flexible than GBrowse and scales easily to multi-gigabase genomes. The input formats for JBrowse are GFF3, BED, FASTA, Wiggle, BigWig and BAM. The architecture of GARfield is shown in Figure S1.

JBrowse allows one to upload, compare and analyze an original reference DNA sequences and set of tracks for describing different features of the genome from different species. The reference sequence of Fca-6.2 genome for the new browser in FASTA format was downloaded from ftp://ftp.ncbi.nlm.nih.gov/genomes/Felis_catus/. To assure the accuracy of the reference, a comparison of the references was made from different sources: [NCBI - http://www.ncbi.nlm.nih.gov/assembly/440818/](http://www.ncbi.nlm.nih.gov/assembly/440818/), [Ensembl - http://www.ensembl.org/Felis_catus/Info/Index](http://www.ensembl.org/Felis_catus/Info/Index), and [UCSC - http://hgdownload.soe.ucsc.edu/goldenPath/felCat5/bigZips/.](file:///C:\Downloads\UCSC%20-%20http:\hgdownload.soe.ucsc.edu\goldenPath\felCat5\bigZips\) Although these sources were different, the source DNA sequences (Fca-6.2) are the same.

A genes track on the GARfield browser includes 22,656 gene regions that were annotated in Ensembl (gene transcripts like coding genes, small non-coding genes, pseudogenes, etc.) [<http://www.ensembl.org/Felis_catus/Info/Index>] (9), but were also validated using a comparative approach that detects gene homology in well annotated mammalian genomes: *Homo sapiens, Pan troglodytes, Mus musculus, Rattus norvegicus, Bos taurus, Canis familiaris, Macaca mulatta, and Equus caballus*. The tracks were preprocessed and converted to GFF3 format with scripts located at http://[GARfield.dobzhanskycenter.org/supplements/index.html](http://garfield2.dobzhanskycenter.org/supplements/index.html). GARfield displays annotated tracks for genes, indels, SNPs, different types of repeats, such as large interspersed repeats, families of complex tandem repeats, short tandem repeats (STRs or microsatellites) and adjacent PCR primer sequences, CpG and non-CpG methylated sites, microRNA sequences, ultra conserved sequences among mammalian genomes, nuclear mitochondrial DNA (Numts), pseudogenes, putative endogenous retroviral elements (ERVs), segmental duplicated regions, an assisted assembly of *Felis silvestris silvestris* plus homologous synteny blocks (HSBs) based upon alignment and analyses with other mammalian genome sequences. Fca-6.2 is anchored to chromosome coordinates with two physical framework maps: 1.) a radiation hybrid map; 2.) STR linkage map (5,6,8,10,11).

GARfield data can be downloaded in FASTA and GFF format, and users can upload their own data for display using the supplemental Graphical User Interface (GUI). An interactive edition of the tracks parameters permits a user to control graphical presentation of genome elements, create new virtual tracks as a combination (union, XOR, subtraction, intersection), mask a track by another tracks and easily scale and highlight area of interests. Virtual rules help to compare relative position of elements. GARfield also includes hyperlinks to the annotated features and related resources on the Internet.

Many GARfield annotations extend the information available from the cat genome browsers at NCBI ([http://www.ncbi.nlm.nih.gov/nuccore/?term=felis catus](http://www.ncbi.nlm.nih.gov/nuccore/?term=felis%20catus)), University of California Santa Cruz (UCSC) (<http://genome.ucsc.edu/cgi-bin/hgGateway?org=Cat>), and Ensembl (<http://www.ensembl.org/Felis_catus/index.html>). First, GARfield allows coordination of tracks and data without limits of the data size or time keeping the data on the server. GARfield also provides a GUI allowing rapid adjustment to meet the specific user-defined requirements. GARfield follows the [GMOD project (http://www.gmod.org/wiki/Main_Page](http://www.gmod.org/wiki/Main_Page)) guidelines as a web-oriented, open source, well supported platform which permits to create a new custom Graphical User Interface.

The annotated features described below are available in GARfield ([http://GARfield.dobzhanskycenter.org](http://garfield2.dobzhanskycenter.org)) and the UCSC Genome Browser (http://genome.ucsc.edu) which links simply to the Dobzhansky Center Hub as follows:

1. Go to <genome.ucsc.edu>
2. Click <Genome Browser> bar
3. Click <Track hubs> bar
4. Copy {<http://public.dobzhanskycenter.ru/Hub/hub.txt>} to URL window
5. Click <Use Selected Hubs>

This reveals tracks in the cat genome.

**III. Gene annotation**

Gene analysis was carried out in two steps. First, reciprocal best matches between the cat genome and reference genomes were analyzed to derive statistics on reference genome gene feature coverage. Second, alignments between reference genome gene exons and the cat genome sequences were inspected to get putative regions for cat genes.

*Reference genomes and their features.* Reference genomes were downloaded from NCBI, their gene annotations were imported from NCBI RefSeq database (12). Gene feature statistics are shown in Table S1. For each gene, the longest mRNA and corresponding coding sequences (CDSs) and exons were chosen for further analysis. Also 3'-UTR, 5'-UTR, 5 kb up- and downstream regions were identified. 5'-UTR regions were identified as the regions between the first exon start and the first CDS start, 3'-UTR regions were identified as the ones between the last CDS end and the last exon end. The cat genome from Fca-6.2 assembly was compared to seven annotated mammalian genomes using a reciprocal best match (RBM) approach. Statistics on the reference genome features used for gene annotation are shown in Table S2.

*Masking of repetitive elements.* Fca-6.2 chromosomes were masked in two different ways. First, repetitive elements were searched for using RepeatMasker 4.0.2 with RepBase Update 20130422 database. RepeatMasker options were the following: *-s -species cat -xsmall -nolow*, which means sensitive search of repetitive regions except for low-complexity regions and masking them with lower-case letters. Second, WindowMasker (13), a *de novo* repeat masking program, was applied to Fca-6.2 assembly using default settings. Finally, a combined masking was constructed from the results of RepeatMasker and WindowMasker in the following way: each nucleotide in combined masking was masked if it had been masked by RepeatMasker or WindowMasker. Reference genome masking was obtained by RepeatMasker from NCBI.

*Chromosome alignment:* NCBI BLAST+ 2.2.25 package (14) was used for chromosome sequence alignment. For each reference genome, BLAST databases containing the sequences and the masking were created. Then each chromosome of Fca-6.2 assembly was aligned to these databases as a query using *blastn* program from the package. Alignment parameters were the following: *-dust yes -soft_masking true -lcase_masking -penalty -1 -reward 1 -gapopen 0 -gapextend 2 -xdrop_gap 40 -word_size 16 -db_soft_mask 40*, which means exact match between two regions of at least 16 bp, enabled soft masking both in query and subject sequences (that is, alignment can expand through the masking, but cannot start in it) and enabled filtering of a query sequence with the build-in DUST module (15) in order to skip low-complexity regions.

*Reciprocal Best Matches (RBMs).* Given a set of pairwise alignments, we stipulate that regions A and B form a reciprocal best match (RBM) if there is no region C that aligned to A with a score higher than B and there is no region D that aligned to B with a score higher than A. From the set of pairwise alignments between the cat genome and the reference genomes, a set of RBMs was derived (Table S3). Values provided are mean and standard deviations of RBM percent identity, length, and relative length (that is, a ratio of length of RBM region in the reference genome to the length of the corresponding region in the cat genome), total number of RBMs and percent of the cat assembly covered by them. For each reference genome, reciprocal best matches were checked if they contained any gene elements within the reference genomes (Table S4).

*Gene detection by exon alignments*. Genes in Fca-6.2 assembly were detected with the comparative approach using eight mammalian genomes (the same ones as for genomes comparison plus horse – EquCab2.0 assembly) with annotations of their protein-coding genes from Ensembl Genes 72 database (16). The Ensebml Gene database was chosen since it explicitly provided access to gene exon sequences and gene, transcript, and exon interrelationship using Biomart interface (17). In Table S5-S7, the numbers of protein-coding genes for reference genomes are shown.

The following procedure was used to find the genes of each reference genome.

1. Exon sequences of protein-coding genes were obtained from Ensembl Gene 72 database.
2. The exon sequences were aligned to the cat chromosomes using blastn tool from NCBI BLAST 2.2.25+ package (14). The chromosomes were masked with combined masking from RepeatMasker and WindowMasker (see subsection 'Masking of repetitive elements' above). Alignment options were the following: *-dust no -word_size 16*.
3. Derived alignments were analyzed for each reference genome transcript. A transcript was considered to be found in the cat genome, if all its exons were found at the same chromosome, their orientation was the same, and the order of exon alignment regions in the cat genome was the same as the order of exons in the transcript.
4. A gene from a reference genome was considered to be present in the cat genome, if any its transcript was detected in the way described in the previous step.

In Table S6, the numbers of genes detected by the described approach are shown. In Table S7, the numbers of detected genes shared between various reference genomes are shown. The total number of the detected genes is 21,865.

**IV. DNA variants**

SNPs and indels in Fca6.2 were derived from 30 whole genome sequences (411 sequence runs in total) from Washington University Genome Sequencing Center deposited in NCBI SRA database. All reads were filtered and clipped using Trim Galore with default parameters. Short reads were aligned to reference Fca6.2 genome using bowtie2 default parameters (bowtie2 -x FelisCatus6.2 - p30 -U raw_reads.fq -S aligned_reads.sam) (18). For SNP calling and VCF-file processing we used the combination of samtools and vcftools (19,20). A total of 211,833 variants were detected after filtering the ones with low quality (Phred score less than 20). Also the variants located in repeat regions were removed, and we obtained list of 99,494 SNPs (53,99% lay in repeat regions). Coordinates of repeat elements were obtained from merging repeats detected by RepeatMasker, WindowMasker and DustMasker (see section V). In total there were 61% homozygous variants (Table S8). Average coverage and quality scores for SNVs and indels after filtering were 6.7 and 39.6, respectively (Table S9). Number of observed variants per chromosome is correlated with chromosome size, the correlation coefficient value is 0.87 (Table S9, Figures S2 and S3).

**V.** **Repeat Content in *Felis catus* genome (Fca-6.2)**

Repetitive Elements (REs) are common residents of nearly all genomes and their amount seems to increase with the genome complexity and size. REs can be divided into two main types: 1.) Interspersed Repeats (IRs, including Transposable Elements (TEs), or transposons) and 2.) Tandem Repeats (TR). TRs usually divided into: a) Complex Tandem Repeats (CTRs, including satellite DNA), and b) Short Tandem Repeats (STRs, also called simple sequence repeats or microsatellites) which are built of 2-7 bp long monomer sequence. TRs are found ubiquitously in genomes of both prokaryotic and eukaryotic organisms. Their density and distribution across the genome is unequal and seemingly non-random. In eukaryotic genomes TRs can be found in introns of protein-coding genes, in centromeric regions (e.g. human alphoid DNA), in telomeres, and also in cystrones of rRNA genes and low-complexity regions (22).

Interspersed Repeats (IRs) are usually 0.1-10 kbp long and represent active TEs or their fragments scattered across the genome. IRs have been found in almost all eukaryotic species studied (23). The principal TE groups are ancient, ubiquitous across kingdoms, and display extreme diversity. Plants usually have the most abundant variety of TEs, although TEs are also widespread across genomes of fungi (5-27% of genome) and animals (3-50% of genome) (24).

Searches across Fca-6.2 were performed with RepeatMasker software (25) using RM-BLAST as a search engine. Repbase Update (version 20130422-2013; <http://www.girinst.org>) was utilized to detect known repeats sequences (26). We ran RepeatMasker with «high sensitivity» option and utilized a library of REs that had been previously described for *F. catus* (with «species cat» option). Masking of the found REs was carried out with «xsmall» options that returned a chromosome's sequence file. RepeatMasker produced 3 output text files for each cat chromosomes:

1. a FASTA file with masked REs;
2. an *annotation file* which contained the cross_match output lines,
3. a *summary file* with the table that depicted absolute and relative contents of the main types and families of REs found in a chromosome.

An annotation file lists all best matches between the cat sequence and Repbase sequences. We illustrate the numbers of different groups and subgroups of REs found in Figures S4 and S5 with REs family length estimates in Table S10.

WindowMasker is a *de novo* repeat finding tool that is based on frequency counts of different k-mers within a nucleotide sequence (13). Unlike RepeatMasker, it does not require any library of repetitive sequences and therefore can be applied to the genomes of species, which have not been investigated yet. We ran WindowMasker version 1.0.0 (<ftp://ftp.ncbi.nlm.nih.gov/blast/executables/blast+/2.2.25>), using its default options. We compared the number of discrete elements, the length occupied by REs on each chromosome and percentage of masked nucleotides per chromosome produced by RepeatMasker and WindowMasker (Table S11). We constructed databases with masking information (RM-repeats) for all discovered REs found in Fca-6.2 by RepeatMasker and WindowMasker.

TRs in Fca-6.2 and in the unplaced contigs (Chromosome Unknown, ChrUn, <ftp://ftp.ncbi.nlm.nih.gov/genomes/Felis_catus/CHR_Un/>) were detected with Tandem Repeats Finder (TRF) software, version 4.07 (27). Search parameters were: mismatch - 5; maximum period size - 2000; other parameters - default. To eliminate any redundant entries from the TRF output, all embedded TR arrays were discarded; if two arrays had the same sequence coordinates a TR with higher variability was discarded. Overlapping arrays were considered as independent arrays. Each TR has several variants of monomer consensus sequences generated by: (1) sequence rotation, (2) presence of reverse complement, and (3) monomer multiplication. We corrected monomer consensus sequences according to the definition of the monomer consensus sequence as a lexicographically minimal sequence from lexicographically sorted rotations of sequence and its reverse complement.

Found TRs were divided into three groups: 1) STRs, 2) CTRs and 3) remaining TRs. Presence of the third group can be explained by high TRs variability and low quality assembly for regions of tandem repeated DNA. CTRs included large tandem repeats and satellite DNA characterized by: GC-content of arrays from 20% to 80%, array length greater than 100 bp, copy number greater than 4, array entropy greater than 1.76, monomer length greater than 4 bp and imperfect TR organization. CTRs were classified into families by sequence similarity computed by Blast program according to the workflow from (28). Each family was named according to nomenclature based on the most frequent monomer length (Figure S7). For visualization, CTRs were plotted according to their GC-content, monomer length, and variability of monomers inside arrays using Mathematica™ 7.0 program. Positions of CTRs on assembled chromosomes were visualized with PyChrDraw program (<https://github.com/ad3002/PyChrDraw>).

Derived repeat family data were confirmed by comparing them with Dustmasker analysis of Fca-6.2 (default options). Dustmasker, available within WindowMasker (-dust option), implements symmetric algorithm for masking of low-complexity regions called «DUST». As CTRs mostly do not have to be masked by Dustmasker, we included them in this comparison. We also added data, which were obtained by RepeatMasker with option “nolow”. This option turns off masking of low-complexity regions and STRs, and provides searching only for IRs and CTRs. (Table S12). REs in the whole genome *F. catus* were previously characterized on 1.9x coverage cat genome assembly (1,29). We confirm and extend these results but depict some inaccuracy of low-coverage assembly in many values characterizing the REs content. Most discrepancies can be explained by low resolution of REs boundaries and older version of Repbase Update, which contained less characterized sequences. In Fca-6.2 ~55.72% of 2.43 Gbp cat genomes (1.32 Gb) were masked as repetitive elements: 39% (963 Mbp) were found as IRs and only less than 4% corresponded to TRs.

*Interspersed Repeats*. RepeatMasker detected 39% of cat genome as IRs (Table S12). The frequent superfamilies of IRs are: LINEs – 20.2% (among them 16.4% belong to LINE/L1 family), SINEs – 11% and LTR elements – 5.03% (including endogenous retroviruses). DNA transposons comprise only 2.75% of full genomic sequence. Absolute numbers of found elements for REs groups are shown in Fig. S4 and revealed the prevalence of SINE/tRNA-Lys family members and LINE/L1 elements.

The X chromosome has the highest repeat content (~50.93% masked) while chromosome E1 and E3 have the lowest (34.47% and 36.63%, respectively) reflecting differences in content of LINE elements. About 32.39% of X chromosome are LINE elements, the highest value for LINEs across all chromosomes, but at the same time chromosome X has a ~10.54% content of SINEs. Chromosome E1 has 12.79% of SINE elements which is the highest content of all chromosomes.

Results of comparison between RM-repeats and WM-repeats in Fca-6.2 are shown in Table S11 and Fig. S6. WindowMasker detected 776 Mbp (~31.61%) of Fca-6.2 as REs. RepeatMasker did not detect 50.33% of WM-repeats (Table S11). WindowMasker tended to miss mostly LINE elements leaving them unmasked.

*Complex Tandem Repeats.* TRs found by TRF were represented by 862,209 arrays with total length of 51.8 Mbp. STRs made up 69.2% of all TRs found (Table S12). CTRs group comprised only 0.3% of all TRs found in Fca-6.2 and 11.2% of all found in ChrUn contigs largely due to unassembled pericentromeric and centromeric regions enriched with satellite DNA (30). RepeatMasker detected 287 discrete elements of CTRs in the whole cat genome that comprised about 0.015% of the genome sequence length (Table S12). To simplify results representation, all single locus families were joined into SL (Single Locus) group and all families with number of arrays less than 6 were joined into ML5 (Multi Locus 5) group (Table S13). The families from WGS assembly with largest arrays were visualized according to their GC-content and monomer similarity in array (Fig. S8). *TR-483A-FC* family is a feline-specific satellite DNA (*FA-SAT*) reported as representing 1–2% of the cat genome (31). We identified more than 25 novel undescribed families of complex tandem repeats in the cat genome (Table S13). *TR-31A-FC*, *TR-31B-FC*, *TF-68A-FC* and *TR-26A-FC* families were found only in ChrUn due to localization in centromeres. Families *FA-SAT* (*TR-483A-FC*), *TR-19A-FC*, and *TR-33A-FC* had more arrays in ChrUn than in assembled chromosomes, and therefore also can be candidates for localization in centromeric or pericentromeric regions. Families with fewer arrays (SL and ML5) were assembled on chromosomes (for single locus repeats: 1,708 arrays on chromosomes and 32 arrays in ChrUn).

When CTRs were mapped on the assembled chromosomes (Fig. S9) their dispersal was seemingly non-random. We also observed an enrichment of telomeric/pre-telomeric regions in cat with low-copy families (Fig. S10-12). The *FA-SAT* family is known as GC-rich, mapped by FISH to telomeric regions, and not present in all cat chromosomes (32). We mapped *FA-SAT* to Fca-6.2 (Fig. S13) and found certain conflicts, namely, *FA-SAT* presence on chromosomes A1 and A2 and absence on chromosomes B2 and F2 predicted by (32). These conflicts may be a signal of misassembles of regions of these chromosomes in Fca-6.2. A correct assembly of large arrays of satellite DNA remains the one of the hardest challenges in genome assembly (1,29).

Since Dustmasker tends to include gaps into its masking, gap regions were excluded from the set of the regions masked by it. This exclusion reduced the total length of the masked regions from 247 Mbp to 157 Mbp and increased the number of masked regions from 4,576,346 to 4,636,620 (about 1.3% from the original number) because some regions were split after the gap removal. Comparison of repeats identified by Dustmasker to the ones found by other tools revealed the following.

1. More than 80% of REs detected by Dustmasker lay within WM-repeats.
2. More than 65% of REs detected by Dustmasker did not overlap with low-complexity regions and STRs detected by RepeatMasker with «noint» option.
3. About 36% of REs detected by Dustmasker lay within and 47% of them did not overlap with IRs detected by RepeatMasker.

The application of library-based methods alone usually underestimates the real content of existing REs in mammalian genomes (33-36). For example, for the initial annotation of the human genome, RepeatMasker detected 49% of the whole sequence as repetitive, while subsequent application of *de novo* searching algorithms revealed that more than 60% of the human genome may be comprise of REs (37). For this reason, we shall concentrate on search approach algorithms that detect previously undiscovered repeats in the cat genome and in genomes of other vertebrates.

*Short Tandem Repeats****.*** RepeatMasker detected a bit less than 1.5 million STRs (totaling 70.3 Mbp in Fca-6.2, 2.9% of the whole genome sequence, Table S15). Chromosome A1 had the most STR elements that together comprised 2.95% of its length (~7 Mbp). We also analyzed TRs that were classified as STRs after filtration step in CTRs analysis. In contrast to the majority of other mammalian genomes, where the most abundant STR is (AC)_n_ (38), the most common motif in cat is (AG)_n_ that was assembled in 120,319 arrays (11.5% of all found TRs). The other large families of STRs observed were (AC)_n_ with 97,777 arrays (9.3% of all found TRs), and (AT)_n_ with 33,810 arrays (3.2% of all found TRs).

To annotate and design PCR primers useful for population and mapping studies in cats, we searched for the “perfect STRs” applying a Perl script to retrieve coordinates of 2-7-mers occurring a minimum of 5 times in tandem (see Table S16). We detected some 823,000 elements, predominantly dimeric monomers, with 10-fold fewer tetrameric STRs and even fewer trimeric STRs. To avoid primer design within REs, the assembly was masked using WindowMasker (13,15), and any masked nucleotides were converted to ‘N’. For each STR, the STR and the 200 bp flanking regions were retrieved from the masked sequence, and were used as input to Primer3 (39). The STR served as a target region and any unmasked sequence served as candidate region for primers to span the target region. The STR was disqualified from primer design if: 1) the flanking regions included a second STR, 2) the flanking regions included a stretch of polyN of more than 5 nucleotides, or 3) the flanking regions had less than 100 unmasked nucleotides. For each designed primer, e-PCR (40) was then used to screen the primers, retaining those that mapped uniquely to the assembly (settings used for e-PCR: *N=2 G=2 T=3 W=9 F=1*). This strategy allowed the design of 53,710 primer pairs, of which 52,343 (97.4%) mapped uniquely to the cat assembly (Table S16). All repeat feature tracks in BED format were uploaded to GARfield [http://GARfield.dobzhanskycenter.org](http://garfield2.dobzhanskycenter.org).

**VI. Evolutionary constrained elements (ECE)**

To identify evolutionary constrained elements (ECEs) in the cat genome, we used ECEs of the human genome, which were initially annotated by detection of constrained 12-mers using SiPhy-omega algorithm in the MultiZ alignment of 29 mammalian genomes, including cat (earlier assembly version Felis_catus 3.0 (1)) (41). We extracted ECEs from the human genome using BEDTools and mapped them to Fca-6.2 genome assembly by NCBI BLAST 2.2.25+ with its default settings (14). Due to BLAST score cutoff, only ECE clusters of length 23 bp and more were transferred to Fca-6.2. Intersection with genomic features was performed using UCSC table browser (http://genome.ucsc.edu/cgi-bin/hgTables).

We transferred 743,362 ECEs with a total length of 70.01 Mbp (Table S17). The average length of elements was 94.2±95.3 bp, the identity between human and cat elements was 93.7±3.7%. We produced the GARfield track from these data. Additional annotation information on each element includes: position in human genome, LOD-score calculated by SiPhy (indicating the power of constraint), BLAST statistics of the alignment of human elements against cat genome (identity percent, number of gaps and mismatches). We annotate only 20% of ECEs (mean length 94 bp versus 36 bp in (41)) and detected 54% of constrained sequence discovered in human genome (70 of 128.8 Mb) covering 2.95% of cat genome.

We studied the positions of ECEs located in cat chromosomes relative to genes annotated by Ensembl (<http://www.ensembl.org/Felis_catus/Info/Annotation>). 31% of ECEs (31% basewise) lay within exons (which represent 2% of cat genome), and 38% (20% basewise) were within introns (30% of cat genome).

Conservative sequence blocks (CSBs) were also detected by intersecting cat genome regions which formed RBMs with the reference genomes (See section III above). A nucleotide was included in a CSB, if it were found as RBM among all reference genomes. Statistics on the detected CSBs for various reference genome groups are given in Table S18.

We compared ECEs with cat chromosomal positions to Conserved Sequence Blocks (CSBs) detected directly in cat genome by the RBM method (see section III). We used CSB data for whole reference genome set (CSB C). We discovered that the majority of ECE sequences lay within the CSBs consistently represented in mammals (66% of elements and 76% of nucleotide sequence) covering 29% of CSB sequence. This overlap reflects the good correspondence between the genome constraint patterns discovered in human genome by sliding-window alignment analysis and in cat genome using reciprocal best matches.

**VII. Feline endogenous retrovirus-like elements**

In order to detect endogenous retrovirus-like elements in the cat genome, a database of complete viral genome sequences and their fragments published at NCBI was created. The basis of the database is a set of complete genome sequences of exogenous retroviruses from RefSeq database (12) which were filtered by the following query: *txid11632[organism:exp]*. Genomes and genome fragments of retroviruses which had not been included in the set were manually downloaded and added to it for comprehensive coverage of retrovirus family. Also a number of well-known endogenous retroviral sequences for mammalian species were manually downloaded from NCBI and added to the set based on published results in this field. The viral sequence set included:

- 3 RD114 complete genome sequences (accession numbers AB559882.1, AB705393.1, and NC_009889.1) and 2 gene sequences of the virus (accession numbers AF155060.1 and AF155061.1);
- 4 Feline Leukemia Virus (FeLV) complete genome sequences (accession numbers AB060732.2, AB672612.1, M18247.1, and NC_001940.1) and 1 gene sequence of the virus (accession number M12500.1);
- 2 endogenous Feline Leukemia Virus (enFeLV) complete genome sequences (accession numbers AY364318.1 and AY364319.1) and 6 gene sequences of the virus (accession numbers L06140.1, M21479.1, M21480.1, M21481.1, M25425.1, and M25582.1);
- 6 endoretrovirus-like (ERV-L) sequences from dog and cat (accession numbers AJ233664.1, AJ233665.1, AJ233666.1, AJ233667.1, AJ233668.1, and AJ233669.1);
- 8 gene sequences of Feline Sarcoma Virus (FeSV) (accession numbers J02086.1, J02087.1, J02088.1, K01643.1, M23024.1, M23025.1, M23026.1, and X00255.1);
- 15 complete genome sequences of other Feline Endogenous RetroViruses (FERV) (accession numbers AB674439.1, AB674440.1, AB674441.1, AB674442.1, AB674443.1, AB674444.1, AB674445.1, AB674446.1, AB674447.1, AB674448.1, AB674449.1, AB674450.1, AB674451.1, AB674452.1, and X51929.1);
- 3 envelope gene sequences (also include LTRs) of Gardner-Arnstein Feline Leukemia Virus B (accession numbers K01209.1, V01172.1, and X00188.1);
- 1 complete genome sequence of Feline Immunodeficiency Virus (FIV) (accession number NC_001482);
- 3 complete genome sequences of Feline Foamy Virus (FFT) (accession numbers AJ564745.1, AJ564746.1, NC_001871.1);
- 24 syncytin-related envelope protein gene sequences of various mammals (accession numbers JN587088.1, JN587089.1, JN587090.1, JN587091.1, JN587092.1, JN587093.1, JN587094.1, JN587096.1, JN587097.1, JN587098.1, JN587099.1, JN587100.1, JN587101.1, JN587102.1, JN587106.1, JN587107.1, JN587108.1, JN587109.1, JN587110.1, JN587111.1, JN587112.1, JN587113.1, JX412969.1, and NG_004112.1).

Sequences from the set described above were aligned to the masked sequences of cat using LASTZ (42). The following LASTZ options were used: *--ambiguous=iupac --coverage=50 --chain --identity=50 --nofilter --match=2,3 --gap=5,2*. These options correspond to chained hits with more than 50% identity and covering at least 50% of original retroviral sequences. Match reward, mismatch and gap penalty parameters were chosen to provide high-identity alignments. In total, 363 kbp of virus-like sequences, which correspond to 130 kbp of the cat genome, were found (see Table S19A). There were 473 alignments, 12 of them corresponded to RD114 and 24 to enFeLV.

For building the phylogenetic tree of the detected endogenous retrovirus-like elements, MEGA5.2.2 package (43) was used. First, sequences corresponding to *pol* genes were extracted from the database of viral sequences using a Biopython (44) script written by the authors. Only sequences that correspond to definitely annotated features were extracted. Second, the *pol* gene sequences were aligned to the cat genome using LASTZ with the following options: *--ambiguous=iupac --coverage=50 --chain --identity=50 --nofilter --match=2,3 --gap=5,2*. Totally 170 kbp of viral *pol* gene-like sequences were detected. There were 327 alignments, 13 of them corresponded to RD114. Statistics on host species of the viruses, which *pol* genes formed the alignments, are given in Table S19B.

The regions in the cat genome that formed alignments were multiply aligned with muscle tool from MEGA5.2.2. Third, the phylogenetic tree (see Figure S18) was constructed from the alignments using the same tool and visualized with the TreeGraph2 (45) and FigTree (46) tools. The tree was build using the neighbor-joining method. The tree groups correspond to the following viral sequences:

- ERV-L Group – ERV-like sequences,
- DERV Groups 1 and 2– Canis familiaris isolate DERV and Ovis aries endogenous virus gamma 8,
- RD114 Group – RD114 clone Fc41 (accession number AF155061.1) and Wooley monkey sarcoma virus (accession number NC_009424.4),
- PERV Groups 1, 2, and 3 – Porcine ERV FPP-1 (accession number AF163265.1),
- HB Group – Human ERV K (accession number JN202403.1) and Baboon ERV strain M7 (accession number D10032.1),
- HPC Group – Human ERV K (accession number DQ166931.1), Porcine ERV class E clone P141 (accession number AF356697.1), and Canis familiaris ERV-L (accession number AJ233665.1),
- HBPC Group - Human ERV K (accession number JN202403.1), Baboon ERV strain M7 (accession number D10032.1), and Canis familiaris ERV-L (accession numbers AJ233665.1, AJ233667.1, and AJ233668.1).

The tracks describing virus-like and viral-pol-like regions were uploaded in GARfield.

**VIII. Methylation sites in the cat genome**

DNA methylation is an epigenetic modification of genomic DNA found in most eukaryotic taxa including mammals in which ~70–80% of CpG dinucleotides are methylated (47,48). Methylation of cytosine bases affects secondary structure of the DNA and thus alters the ability of chromatin-binding proteins such as transcription factors to attach to their targets. Methylation within promoter regions usually silences transcription and represses gene expression. Methylation accumulates during somatic development, although external stimuli can cause either the methylation or demethylation of specific sites. Differentially methylated regions (DMRs) have been identified in many species, developmental stages and cancer types as being involved in tissue-, cell- or cancer-specific gene expression. To date, it remains largely unknown how patterns of DNA methylation differ between closely related species and whether such differences contribute to species-specific phenotypes (49). Recently, several efficient specialized protocols to identify the unmethylated and methylated regions by measuring the methylation status of cytosines based on the reliable bisulfite sequencing data has been developed (47,48,50-52). We used these techniques in combination with the whole genome sequencing to identify methylated sites in the genome of a domestic cat.

Genomic DNA from blood of mixed breed domestic cat living in St. Petersburg (Russia) was isolated by AxyPrep Multisource Genomic DNA Miniprep kit (Axygen Biosciences). The further workflow for DNA library construction was as follows:

1）Fragmentation of genome DNA to 100-300 bp by sonication;

2）DNA-end repair, 3'-dA overhang and ligation of methylated sequencing adaptors;

3）Bisulfite treatment by ZYMO EZ DNA Methylation-Gold kit;

4）Desalting, size selection, PCR amplification and size selection again;

5）Establishment of qualified library for sequencing.

Data from two libraries with 20x coverage (bisulfite-treated and untreated libraries) were used to perform standard bioinformatics analysis, namely filter data (remove adaptor sequences, contamination and low quality reads), read alignment, sequence depth and coverage analysis.

We implemented a version of the BS-Seeker2 protocol that utilizes a fast short read aligner, Bowtie2, to perform the three-letter alignments (53). The workflow included 3 steps as building the reference genome, mapping to the reference with Bowtie2, and calling methylation. The output files were CGmap, ATCGmap and wig files, the latter one being a wiggle file used for visualizing in a browser. The CGmap produces a numeric call per site as to the number of reads that gave a methylated call (mC) vs the total number of reads (mC + C). It also gives information regarding the methylation coefficient per site = #mC/(mC+C). This is the numeric value per site regarding its methylation status (Table S20).

The cumulative distribution of effective sequencing depth in cytosine was checked and the relationship between genome coverage and read depth was identified. We calculated the methylation coefficient per chromosome  #mC/(mC+C), where mC is a quantity of methylated cytosines and C is amount of unmethylated cytosines. The data show that 10.5% of cytosines of the whole genome are methylated. Distribution of methylated cytosines per chromosome is approximately equivalent between the chromosomes fluctuating from 3.04% in X chromosome to 5.75% in E1 and 6.23% in chromosome E3.

**IX. miRNA**

To locate potential micro-RNA sequences in Fca-6.2 assembly, nucleotide sequences from miRBase (54), containing microRNA elements from 36 species , were aligned to the cat genome masked with RepeatMasker 4.0.2 (25) program and Repbase Update database (26) release 20130422 using **blastn** tool from NCBI BLAST+ 2.2.25 package (55). RepeatMasker was used with the following options: *-s -species cat -nolow*, which correspond to sensitive search for cat-specific repeats without masking low-complexity regions. blastn was used with the following options: *-word_size 16 -penalty -1 -reward 1 -gapopen 0 -gapextend 2 -dust yes*, which require exact match of at least 16 nucleotides between sequences, set on low-complexity masking of micro-RNA sequences, and specify alignment parameters that allow short gaps. A total of 19,071 alignments between the micro-RNA sequences and the cat genome were identified. Then the alignments that had an e-value more that 10^-5^, length less than 50 bp, or identity less that 95% were excluded, and the number of alignments reduced to 3,182. For those alignments, the corresponding regions from the cat genome were extracted and processed with RNAfold program (56) to determine minimum free energy (MFE) of secondary structure. We also used RNAfold to collect information about MFE of all entries in miRBase database. An alignment was considered to be a putative miRNA if its MFE was in range of MFE’s from miRBase. Data were added to GARfield browser as a separate track. In sum we annotated 3,182 feline miRNA homologues in Fca-6.2 based upon matching miRNA from 36 vertebrate species (Table S21).

**X. Nuclear mitochondrial segments (*Numts*) in Fca-6.2**

BLAST searches performed with the whole *Felis catus* cytoplasmic mtDNA genome (NC_001700) used as a query sequence against Fca-6.2 retrieved 430 hits or 174,876 bp of homologues sequences covering 100% of the mtDNA genome. We retrieved hits covering ~96% of the previously described 7.8 kbp Lopez-*numt*, which was observed to be tandemly repeated 38-76 times on the domestic cat chromosome D2 and annotated in the 1.9x coverage of the *F. catus* genome (57-59).

Here we discover and map distinct *numts* located on most of cat chromosomes suggesting multiple, independent historic *numt* nuclear insertions covering different regions of the mitochondrial genome. Approximately 15% of the *numts* (<40,000 bp of *numts*) detected in 1.9x coverage of the *F. catus* genome could be mapped to cat chromosomes due to the absence or reduced coverage of *numt*-nuclear junctions (1,59) For Fca-6.2 it has been possible to map 174,876 bp of *numts* providing a much clearer catalogue of *numts* in the cat genome. All cat chromosomes with the exception of chromosome E1 showed evidence of *numts*, with more than 20,000 bp of *numts* found in chromosome A1, more than 15,000 bp of *numts* found in chromosome B4, D2 and X, and another nine chromosomes showing between 15,000 to 5,000 bp of *numts* (Fig. S14). In addition, large *numts* (> 1,000 bp) were detected in 14 of the 19 cat chromosomes, including *numts* comparable in size to the larger 7.8 kbp Lopez-*numt* in chromosome D2, such as a 6.9 kbp *numt* in chromosome B4, a 4.4 kbp *numt* in chromosome D4, a 4.3 kbp *numt* in chromosome A1 and a 4.0 kbp *numt* in chromosome D1. Such large *numts* can confound the analyses of mtDNA in the domestic cat and further analyses are in progress to determine if they are independent insertions or if they may result from secondary integrations (i.e. from the larger 7.8 kbp Lopez-*numt* in chromosome D2).

**XI. Segmental duplications in the domestic cat genome**

Regions of recent autosomal segmental duplications were estimated across the domestic cat Fca-6.2 assembly using the re-sequenced genome with Illumina technology taking advantage of the differences in the depth of coverage (60,61) and the resulting coordinates were included in GARfield. In short, the original 100-bps Illumina reads were clipped into 36-bps high quality reads after trimming the first 10 bps to avoid lower-quality positions. As a result, a total of 1,485,609,004 reads for mapping (coverage = 21.8X) were used (Table S22).

We downloaded the Fca-6.2 (UCSC felCat5) assembly from The UCSC Genome Browser (<http://genome.ucsc.edu/>). The 5,480 scaffolds that were either unplaced or labeled as random were concatenated into a single artificial chromosome. In addition to the repeats already masked in felCat5 with RepeatMasker (www.repeatmasker.org) and Tandem Repeats Finder (27), we sought to identify and mask potential hidden repeats in the assembly. In order to do so, chromosomes were partitioned into 36-bps k-mers (with adjacent k-mers overlapping 5 bps) and these were mapped against the assembly using mrsFast (62) (Figure S15).

*Mapping and copy number estimation from read depth.* The Illumina 36-bps reads resulting from clipping the original FASTQ reads (see above) were mapped to the prepared reference assembly using mrFast (60). mrCaNaVaR (version 0.41) (60) was used in order to estimate the copy number along the genome from the mapping read depth. Briefly, mean read depth per base pair is calculated in 1-Kbps non-overlapping windows of non-masked sequence (that is, the size of a window will include any repeat or gap and thus the real window size may be larger than 1 Kbps). Importantly, because reads will not map to positions covering regions masked in the reference assembly, read depth will be lower at the edges of these regions, which could underestimate the copy number in the subsequent step. To avoid this, the 36 bps flanking any masked region or gap were masked as well and thus not included within the defined windows. In addition, gaps >10 Kbps were not included within the defined windows. A read depth distribution is obtained through iteratively excluding windows with extreme read depth values relative to the normal distribution and the remaining windows are defined as control regions (Table S23). The mean read depth in these control regions is considered to correspond to copy number equal to two and used to convert the read depth value in each window into a GC-corrected absolute copy number. Of the 993,102 control windows, none laid on the artificial chromosome (see above) and 37,123 (3.7%) were on chromosome X.

*Characterization of duplications and deletions****.*** We used a conservative approach to annotate the segmental duplications in the cat autosomes. The copy number distribution in the control regions was used in order to define sample specific gain/loss cutoffs as the mean copy number plus/minus three units of standard deviation (calculated not considering those windows exceeding the 1% highest copy number value). Note that as the mean copy number in the control regions is equal to two by definition, the gain/loss cutoffs will be largely influenced by the standard deviation. Then, we merged 1-Kbps windows with copy number larger than sample-specific gain cutoff (but lower than 100 copies) and identified as duplications the regions that comprised at least five 1-Kbps windows and >10 Kbps. Finally, only duplications with >85% of their size not overlapping with repeats were retained.

We estimated the copy number genome wide in the 1-Kbps non-overlapping windows (Table S22, Figure S16) and illustrated the distribution of duplications by chromosome in Figure S17.

**XII. Assisted assembly of *Felis silvestris silvestris* genome**

To investigate genome variations in European wildcat, *Felis silvestris silvestris*, we used a combination of tools (bowtie2, samtools, vcftools) that was also used for assessing variance in *Felis catus* genome. A 200-fold whole genome sequence coverage or short SOLiD reads across a, *Felis silvestris silvestris,* was mapped by bowtie2 to reference cat chromosomes (Fca-6.2). A total of 380 million reads were aligned to the Fca-6.2 genome. Average coverage for observed variants was 55X (minimum 2X, median 49X). In total we found 2,847,548 single nucleotide variants and 473,887 insertion-deletion variants between domestic cat and wildcat. All polymorphic and fixed difference variants (between Fca6.2 and *F. silvestris*) were added to GARfield.

Among all variants 24.6% (693,428 SNVs and 122,333 indels) were heterozygous in *Felis silvestris*. Between the genomes of *Felis catus* and *Felis silvestris* some 2.9 million (2,847,548) single nucleotide variants and ∼1.9 Mbp of insertions and deletions were detected and annotated in GARfield. Observed differences were significantly fewer compared to difference between human and chimpanzee genomes (~35 million SNV and ~90 Mbp of indels) (63).

**REFERENCES**

References Cited

1. Pontius JU, Mullikin JC, Smith DR; Agencourt Sequencing Team, Lindblad-Toh K, Gnerre S, Clamp M, Chang J, Stephens R, Neelam B, Volfovsky N, Schäffer AA, Agarwala R, Narfström K, Murphy WJ, Giger U, Roca AL, Antunes A, Menotti-Raymond M, Yuhki N, Pecon-Slattery J, Johnson WE, Bourque G, Tesler G; NISC Comparative Sequencing Program, O'Brien SJ: **Initial sequence and comparative analysis of the cat genome.** *Genome Res* 2007, **17(11)**:1675-1689.
2. Mullikin JC, Hansen NF, Shen L, Ebling H, Donahue WF, Tao W, Saranga DJ, Brand A, Rubenfield MJ, Young AC, Cruz P; NISC Comparative Sequencing Program, Driscoll C, David V, Al-Murrani SW, Locniskar MF, Abrahamsen MS, O'Brien SJ, Smith DR, Brockman JA: [**Light whole genome sequence for SNP discovery across domestic cat breeds.**](http://www.ncbi.nlm.nih.gov/pubmed/20576142) *BMC Genomics* 2010, **11**:406.
3. Hillier LW, Warren W, O’Brien SJ ,Wilson RK, International Cat Genome Sequencing Consortium. **NCBI** [http://www.ncbi.nlm.nih.gov/nuccore/AANG00000000]
4. Miller JR, Delcher AL, Koren S, Venter E, Walenz BP, Brownley A, Johnson J, Li K, Mobarry C, Sutton G: **Aggressive assembly of pyrosequencing reads with mates.** *Bioinformatics* 2008, **24**:2818-2824.
5. Davis BW, Raudsepp T, Pearks Wilkerson AJ, Agarwala R, Schäffer AA, Houck M, Chowdhary BP, Murphy WJ: **A high-resolution cat radiation hybrid and integrated FISH mapping resource for phylogenomic studies across Felidae.** *Genomics* 2009, **93**:299-304.
6. Menotti-Raymond M, David VA, Schäffer AA, Tomlin JF, Eizirik E, Phillip C, Wells D, Pontius JU, Hannah SS, O'Brien SJ: **An autosomal genetic linkage map of the domestic cat, Felis silvestris catus.** *Genomics* 2009, **93**:305-13..
7. Stein LD, Mungall C, Shu S, Caudy M, Mangone M, Day A, Nickerson E, Stajich JE, Harris TW, Arva A, Lewis S: **The generic genome browser: a building block for a model organism system database.** *Genome Res* 2002, **12**:1599-1610.
8. Pontius JU, O'Brien SJ: **Genome Annotation Resource Fields--GARFIELD: a genome browser for Felis catus.** *J Hered* 2007, **98(5)**:386-389.
9. Flicek P, Ahmed I, Amode MR, Barrell D, Beal K, Brent S, et al: **Ensembl 2013.** *Nucleic acids research,* **41(D1)**: D48-D55.
10. Murphy WJ, Davis B, David VA, Agarwala R, Schäffer AA, Pearks Wilkerson AJ, Neelam B, O'Brien SJ, Menotti-Raymond M: **A 1.5-Mb-resolution radiation hybrid map of the cat genome and comparative analysis with the canine and human genomes.** *Genomics* 2007, **89(2)**:189-196.
11. Lewin HA, Larkin DM, Pontius J, O'Brien SJ: **Every genome sequence needs a good map.** *Genome Res* 2009, **19(11)**:1925-1928.
12. Pruitt KD, Tatusova T, Brown GR, Maglott DR: **The Reference Sequence (RefSeq) Database.** In *The NCBI Handbook [Internet]. Chapter* *18*. Edited by McEntyre J, Ostell J. Bethesda (MD): National Center for Biotechnology Information (US); 2002. [<http://www.ncbi.nlm.nih.gov/books/NBK21091/>]
13. Morgulis A, Gertz EM, Schäffer AA, Agarwala R: **WindowMasker: window-based masker for sequenced genomes.** *Bioinformatics* 2006, **22(2)**:134-141.
14. Zhang Z, Schwartz S, Wagner L, Miller W: **A greedy algorithm for aligning DNA sequences.** *Journal of Computational biology* 2000, **7(1-2)**:203-214.
15. Morgulis A, Gertz EM, Schäffer AA, AgarwalaR: **A fast and symmetric DUST implementation to mask low-complexity DNA sequences**. *Journal of Computational Biology* 2006, **13(5)**:1028-1040.
16. Hubbard T, Barker D, Birney E, Cameron G, Chen Y, Clark L, Cox T, Cuff J, Curwen V, Down T, Durbin R, Eyras E, Gilbert J, Hammond M, Huminiecki L, Kasprzyk A, Lehvaslaiho H, Lijnzaad P, Melsopp C, Mongin E, Pettett R, Pocock M, Potter S, Rust A, Schmidt E, Searle S, Slater G, Smith J, Spooner W, Stabenau A, et al: **The Ensembl genome database project.** *Nucleic acids research* 2002, **30**: 38-41.
17. Kinsella RJ, Kähäri A, Haider S, Zamora J, Proctor G, Spudich G, Almeida-King J, Staines D, Derwent P, Kerhornou A, Kersey P, Flicek P: **Ensembl BioMarts: a hub for data retrieval across taxonomic space.** *Database (Oxford)* **2011**:bar030.
18. Langmead B, Salzberg S: **Fast gapped-read alignment with Bowtie 2.** *Nat Methods* 2012, **9**:357-359.
19. Li H, Handsaker B, Wysoker A, Fennell T, Ruan J, Homer N, Marth G, Abecasis G, Durbin R; 1000 Genome Project Data Processing Subgroup: **The Sequence alignment/map (SAM) format and SAMtools.** *Bioinformatics* 2009, **25**:2078-1079.
20. Danecek P, Auton A, Abecasis G, Albers CA, Banks E, DePristo MA, Handsaker RE, Lunter G, Marth GT, Sherry ST, McVean G, Durbin R; 1000 Genomes Project Analysis Group: **The variant call format and VCFtools.** *Bioinformatics* 2011, **27**:2156-8.
21. Meyer LR, Zweig AS, Hinrichs AS, Karolchik D, Kuhn RM, Wong M, Sloan CA, Rosenbloom KR, Roe G, Rhead B, Raney BJ, Pohl A, Malladi VS, Li CH, Lee BT, Learned K, Kirkup V, Hsu F, Heitner S, Harte RA, Haeussler M, Guruvadoo L, Goldman M, Giardine BM, Fujita PA, Dreszer TR, Diekhans M, Cline MS, Clawson H, et al: **The UCSC Genome Browser database: extensions and updates 2013.** *Nucleic Acids Res* 2013, **41**:D64-D69.
22. Cavagnaro PF, Senalik DA, Yang L, Simon PW, Harkins TT, Kodira CD, Huang S, Weng Y: **Genome-wide characterization of simple sequence repeats in cucumber (Cucumis sativus L.).***BMC Genomics* 2010, **11**:569.
23. Wicker T, Narechania A, Sabot F, Stein J, Vu GTH, Graner A, Ware D, Stein N: **Low-pass shotgun sequencing of the barely genome facilitates rapid identification of genes, conserved non-coding sequences and novel repeats.** *BMC Genomics* 2008, **9**:518.
24. Deininger P, Moran J, Batzer M, Kazazian H: **Mobile elements and mammalian genome evolution.** *Curr Opin Genet Dev* 2003*,* **13**:651-658.
25. Smit AFA, Hubley R, Green P (1996-2010): **RepeatMasker Open-4.0.0.** [http://www.repeatmasker.org]
26. Jurka J, Kapitonov VV, Pavlicek A, Klonowski P, Kohany O, Walichiewicz J: **Repbase Update, a database of eukaryotic repetitive elements.** *Cytogentic and Genome Research* 2005, **110**:462-467.
27. Benson G: **Tandem repeats finder: a program to analyze DNA sequences.** *Nucleic Acids Research* 1999*,* **27(2)**: 573-580.
28. Komissarov AS, Gavrilova EV, Demin SJ, Ishov AM, Podgornaya OI: **Tandemly repeated DNA families in the mouse genome.** *BMC genomics* 2011, **12**:531.
29. Pontius JU, O'Brien SJ: **Artifacts of the 1.9x feline genome assembly derived from the feline-specific satellite sequence.** *J Hered* 2009, **100 Suppl 1**:S14-8.
30. Alkan C, Cardone MF, Catacchio CR, Antonacci F, O'Brien SJ, Ryder OA, Purgato S, Zoli M, Della Valle G, Eichler EE, Ventura M: **Genome-wide characterization of centromeric satellites from multiple mammalian genomes.***Genome Res*2011, **21**:137-145.
31. Fanning TG: **Origin and evolution of a major feline satellite DNA.** *Journal of Molecular Biology* 1987, ***197***(4): 627–634.
32. Santos S, Chaves R, Guedes-Pinto H: **Chromosomal localization of the major satellite DNA family (FA-SAT) in the domestic cat.** *Cytogenetic and genome research* 2004, **107(1-2)**:119–22.
33. Edgar R, Myers E: **PILER: identification and classification of genomic repeats**. *Bioinformatics* 2005*,* **21(Suppl 1)**:i152-i158.
34. Price A, Jones N, Pevzner P: **De novo identification of repeat families in large genomes**. *Bioinformatics* 2005*,* **21(Suppl 1)**:i351-358.
35. Gu W, Castoe T, Hedges D, Batzer M, Pollock D: **Identification of repeat structure in large genomes using repeat probability clouds**. *Anal Biochem* 2008*,* **380:**77-83.
36. Saha S, Bridges S, Magbanua Z, Peterson D: **Computational Approaches and Tools used in identification of dispersed repetitive DNA sequences**. *Tropical Plant Biol* 2008*,***1**:85-96.
37. De Koning AP, Gu W, Castoe TA, Batzer MA, Pollock DD: **Repetitive elements may comprise over two-thirds of the human genome.** *PLoS Genet* 2011, ***7***(12), e1002384.
38. Mayer C, Leese F, Tollrian R: **Genome-wide analysis of tandem repeats in Daphnia pulex--a comparative approach.** *BMC Genomics* 2010, **11**:277.
39. Rozen S, Skaletsky H: **Primer3 on the WWW for general users and for biologist programmers.** In *Bioinformatics Methods and Protocols: Methods in Molecular Biology. Volume 132.* Edited by Krawetz S, Misener S. Totowa, NJ: Humana Press; 2000: 365-386. [http://primer3.sourceforge.net/releases.php]
40. Schuler GD: **Sequence mapping by electronic PCR.** *Genome Res* 1997, **7(5)**:541-50. [http://www.ncbi.nlm.nih.gov/sutils/e-pcr/]
41. Lindblad-Toh K, Garber M, Zuk O, Lin MF, Parker BJ, Washietl S, Kheradpour P, Ernst J, Jordan G, Mauceli E, Ward LD, Lowe CB, Holloway AK, Clamp M, Gnerre S, Alföldi J, Beal K, Chang J, Clawson H, Cuff J, Di Palma F, Fitzgerald S, Flicek P, Guttman M, Hubisz MJ, Jaffe DB, Jungreis I, Kent WJ, Kostka D, Lara M: [**A high-resolution map of human evolutionary constraint using 29 mammals.**](http://www.ncbi.nlm.nih.gov/pubmed/21993624) *Nature* 2011, **478**:476-482.
42. Harris RS: **Improved pairwise alignment of genomic DNA.** *Ph.D. Thesis*. The Pensylvania State University; 2007.
43. Tamura K, Peterson D, Peterson N, Stecher G, Nei M, and Kumar S **MEGA5: Molecular Evolutionary Genetics Analysis using Maximum Likelihood, Evolutionary Distance, and Maximum Parsimony Methods.** *Molecular Biology and Evolution* 2011, **28**: 2731-2739.
44. Cock PJ, Antao T, Chang JT, Chapman BA, Cox CJ, Dalke A, Friedberg I, Hamelryck T, Kauff F, Wilczynski B, de Hoon MJ: **Biopython: freely available Python tools for computational molecular biology and bioinformatics.** *Bioinformatics* 2009, **25(11)**, 1422–1423.
45. Stover BC, Muller KF: **TreeGraph 2: Combining and visualizing evidence from different phylogenetic analyses.** *BMC Bioinformatics* 2010, **11**:7.
46. **FigTree: a graphical viewer of phylogenetics trees** [http://tree.bio.ed.ac.uk/software/figtree/]
47. Bird A, Taggart M, Frommer M, Miller OJ, Macleod D: **A fraction of the mouse genome that is derived from islands of nonmethylated, CpG-rich DNA.** *Cell* 1985, **40**:91–99.
48. Suzuki MM, Bird A: **DNA methylation landscapes: provocative insights from epigenomics.** *Nat Rev Genet* 2008, **9(6)**:465-76.
49. Zeng J, Konopka G, Hunt BG, Preuss TM, Geschwind D, Yi SV: **Divergent Whole-Genome Methylation Maps of Human and Chimpanzee Brains Reveal Epigenetic Basis of Human Regulatory Evolution.** *The American Journal of Human Genetics* 2012, **91**: 455–465.
50. Feng S, Rubbi L, Jacobsen SE, Pellegrini M: **Determining DNA Methylation Profiles using sequencing.** *Methods of Molecular Biology* 2011, **733**: 223-238.
51. Su J, Yan H, Wei Y, Liu H, Liu H, Wang F, Lv J, Wu Q, Zhang Y: **CpG_MPs: identification of CpG methylation patterns of genomic regions from high-throughput bisulfite sequencing data.** *Nucleic Acids Res* 2013, **41(1)**:e4.
52. Souaiaia T, Zhang Z, Chen T: **FadE: whole genome methylation analysis for multiple sequencing platforms.** *Nucleic Acids Res* 2013, **41(1)**:e14.
53. Guo W, Fiziev P, Yan W, Cokus S, Sun X, Zhang MQ, Chen PY, Pellegrini M: **BS-Seeker2: a versatile aligning pipeline for bisulfite sequencing data.** *BMC Genomics* 2013, **14(1)**:774.
54. Griffiths-Jones S, Grocock RJ, van Dongen S, Bateman A, Enright AJ: **miRBase: microRNA sequences, targets and gene nomenclature.** *Nucleic Acids Res* 2006, **34**:D140-144.
55. Altschul SF, Madden TL, Schäffer AA, Zhang J, Zhang Z, Miller W, Lipman DJ: **Gapped BLAST and PSI-BLAST: a new generation of protein database search programs.** *Nucleic Acids Res* 1997, **25(17)**:3389-3402.
56. Hofacker IL, Stadler PF: **Memory efficient folding algorithms for circular RNA secondary structures.** *Bioinformatics* 2006, **22(10)**:1172-1176.
57. Lopez JV, Yuhki N, Masuda R, Modi W, O'Brien SJ: **Numt, a recent transfer and tandem amplification of mitochondrial DNA to the nuclear genome of the domestic cat.** *J Mol Evol* 1994, **39**:174-190.
58. Lopez JV, Cevario S, O'Brien SJ: **Complete nucleotide sequences of the domestic cat (Felis catus) mitochondrial genome and a transposed mtDNA tandem repeat (Numt) in the nuclear genome.** *Genomics* 1996, **33**:229-246.
59. Antunes A, Pontius J, Ramos MJ, O’Brien SJ, Johnson WE: **Mitochondrial introgressions into the nuclear genome of the domestic cat.** *J Hered* 2007, **98**:414-420.
60. Alkan C, Kidd JM, Marques-Bonet T, Aksay G, Antonacci F, Hormozdiari F, Kitzman JO, Baker C, Malig M, Mutlu O, Sahinalp SC, Gibbs RA, Eichler EE: **Personalized copy number and segmental duplication maps using next-generation sequencing.** *Nat Genet* 2009, **41(10)**:1061-1067.
61. Bailey JA, Gu Z, Clark RA, Reinert K, Samonte RV, Schwartz S, Adams MD, Myers EW, Li PW, Eichler EE: **Recent segmental duplications in the human genome.** *Science* 2002, **297(5583)**: 1003–1007.
62. Hach F, Hormozdiari F, Alkan C, Hormozdiari F, Birol I, Eichler EE, Sahinalp SC: **mrsFAST: a cache-oblivious algorithm for short-read mapping.** *Nat Methods* 2010, **7**:576–7.

SUPPLEMENTAL TABLES

Table S1. Gene and transcript counts for reference mammalian genomes from NCBI RefSeq database (12)

| **Species** | **Assembly** | **Gene** | **mRNA** | **CDS** | **Exon** |
| --- | --- | --- | --- | --- | --- |
| Dog | CanFam3.1 | 24,448 | 21,953 | 225,224 | 241,328 |
| Human | GRCh37.p10 | 41,795 | 37,981 | 381,515 | 457,167 |
| Mouse | GRCm38.p1 | 37,735 | 29,595 | 276,787 | 316,623 |
| Macaque | Mmul_051212 | 32,003 | 29,746 | 257,765 | 301,868 |
| Chimpanzee | Pan_troglodytes-2.1.4 | 33,035 | 34,724 | 312,467 | 362,915 |
| Rat | Rnor_5.0 | 31,618 | 23,991 | 209,058 | 233,606 |
| Cow | Bos_taurus_UMD_3.1 | 27,144 | 22,064 | 200,356 | 222,339 |
| Cat | Felis_catus-6.2 | 22,079 | 21,499 | 228,976 | 243,440 |

Table S2. Gene and gene feature counts for mammalian reference genomes used in the gene annotation procedure. Counts were limited to the genes with the longest mRNA and corresponding coding sequences (CDSs) plus exons.

| **Species** | **Gene** | **mRNA** | **CDS** | **Exon** | **3' UTR** | **5' UTR** | **Downstream** | **Upstream** |
| --- | --- | --- | --- | --- | --- | --- | --- | --- |
| Dog | 24,448 | 19,164 | 187,833 | 191,593 | 19,164 | 9,317 | 24,322 | 24,333 |
| Human | 41,795 | 21,740 | 198,559 | 209,487 | 21,730 | 20,350 | 41,697 | 40,459 |
| Mouse | 37,735 | 23,314 | 201,843 | 212,228 | 23,309 | 20,214 | 37,670 | 36,431 |
| Macaque | 32,003 | 22,575 | 186,165 | 195,008 | 22,573 | 16,009 | 29,912 | 26,994 |
| Chimpanzee | 33,035 | 22,151 | 191,787 | 202,825 | 22,151 | 18,106 | 32,005 | 26,605 |
| Rat | 31,618 | 23,039 | 195,463 | 204,123 | 23,022 | 16,085 | 31,461 | 31,267 |
| Cow | 27,144 | 21,343 | 191,002 | 198,375 | 21,324 | 14,892 | 27,033 | 27,040 |
| Cat | 22,079 | 17,994 | 183,294 | 186,346 | 17,994 | 8,698 | 22,074 | 22,064 |

Table S3. Reciprocal best matches between cat and reference mammalian genomes

| **Species** | **Percent Identity** | **Length** | **Relative Length** | **# RBM** | **% of Cat Assembly** |
| --- | --- | --- | --- | --- | --- |
| Human | 73.3 +/-- 4.4 | 1,483 +/-- 1,218 | 1.0048 +/-- 0.0328 | 657,929 | 38.05% |
| Chimp | 73.1 +/-- 4.5 | 1,367 +/-- 1,152 | 1.0048 +/-- 0.0342 | 756,004 | 40.31% |
| Mouse | 71.0 +/-- 6.1 | 1,059 +/-- 917 | 0.9831 +/-- 0.0327 | 277,028 | 11.54% |
| Rat | 70.8 +/-- 6.2 | 1,011 +/-- 882 | 0.9816 +/-- 0.0330 | 288,586 | 11.49% |
| Dog | 78.9 +/-- 4.7 | 1,468 +/-- 1,370 | 0.9984 +/-- 0.0321 | 1,079,904 | 62.54% |
| Cow | 73.7 +/-- 4.6 | 1,261 +/-- 1,064 | 0.9958 +/-- 0.0342 | 759,885 | 37.58% |
| Macaque | 72.8 +/-- 4.5 | 1,332 +/-- 1,127 | 1.0043 +/-- 0.0346 | 760,387 | 39.49% |

Table S4. Percent representation of reference mammalian genome features in cat RBMs.

| **Species** | **Gene** | **Exon** | **CDS** | **3' UTR** | **5' UTR** | **Downstream** | **Upstream** |
| --- | --- | --- | --- | --- | --- | --- | --- |
| Dog | 86.58% | 92.19% | 92.32% | 85.14% | 90.12% | 94.06% | 94.29% |
| Human | 64.33% | 82.13% | 83.04% | 76.50% | 76.02% | 67.40% | 68.79% |
| Mouse | 60.68% | 68.63% | 70.15% | 58.81% | 60.62% | 46.17% | 46.25% |
| Macaque | 83.06% | 87.40% | 88.08% | 80.83% | 84.63% | 82.88% | 83.60% |
| Chimpanzee | 79.43% | 85.53% | 86.26% | 79.26% | 81.70% | 77.98% | 78.83% |
| Rat | 65.89% | 69.05% | 70.03% | 58.92% | 65.62% | 50.45% | 50.75% |
| Cow | 81.96% | 86.68% | 87.18% | 78.46% | 83.91% | 82.20% | 81.77% |

Table S5 Numbers of protein-coding genes and their transcripts in the reference genomes and the cat genome from Ensembl Genes 72 database (16). Assembly names are given according to NCBI Genome database.

| **Species** | **Assembly** | **# Protein-Coding Genes** | **# Corresponding Transcripts** |
| --- | --- | --- | --- |
| Dog | CanFam3.1 | 19,856 | 25,160 |
| Human | GRCh37.p10 | 22,665 | 159,194 |
| Mouse | GRCm38.p1 | 22,709 | 75,125 |
| Macaque | Mmul_051212 | 21,905 | 36,384 |
| Chimpanzee | Pan_troglodytes-2.1.4 | 18,759 | 19,907 |
| Rat | Rnor_5.0 | 22,941 | 25,725 |
| Cow | Bos_taurus_UMD_3.1 | 19,994 | 22,118 |
| Horse | EquCab2.0 | 20,449 | 22,654 |
| Cat | Felis_catus-6.2 | 19,493 | 20,259 |

Table S6. Counts of cat protein-coding genes that matched gene features of the reference genomes and their transcripts in Ensembl.

| **Species** | **# Protein-Coding Genes Detected** | **# Corresponding Transcripts Detected** | **% Protein-Coding Genes Detected** | **% Corresponding Transcripts Detected** |
| --- | --- | --- | --- | --- |
| Dog | 11,176 | 12,181 | 56.29% | 48.41% |
| Human | 15,300 | 47,707 | 67.50% | 29.97% |
| Mouse | 8,873 | 14,154 | 39.07% | 18.84% |
| Macaque | 8,415 | 10,223 | 38.42% | 28.10% |
| Chimpanzee | 6,061 | 6,191 | 32.31% | 31.10% |
| Rat | 5,589 | 5,713 | 24.36% | 22.21% |
| Cow | 7,255 | 7,478 | 36.29% | 33.81% |
| Horse | 9,885 | 10,149 | 48.34% | 44.80% |

Table S7. The number of genes shared between the cat genome and the reference genomes.

| **# Reference Genomes Genes Are Shared Between** | **# Genes** |
| --- | --- |
| 1 | 10,702 |
| 2 | 3,601 |
| 3 | 2,969 |
| 4 | 2,369 |
| 5 | 1,564 |
| 6 | 660 |
| Total | 21,865 |

Table S8. Detected SNV and Indel genotypic counts for the domestic cat genome.

|  | Homozygous | Heterozygous | Total |
| --- | --- | --- | --- |
| SNV | 59,695 | 39,799 | 99,494 |
| Indel | 6,169 | 2,186 | 8,355 |
| Total | 65,864 | 41,985 | 107,849 |

Table S9. SNV and Indel coverage and counts per cat chromosome

| Chromosome | Average quality score | Median coverage | SNV | Indel |
| --- | --- | --- | --- | --- |
| A1 | 32.8 | 3.05 | 8,300 | 792 |
| A2 | 33.8 | 5.99 | 6,226 | 552 |
| A3 | 33.8 | 3.67 | 7,946 | 610 |
| B1 | 33.5 | 3.84 | 7,654 | 646 |
| B2 | 34 | 3.15 | 6,804 | 494 |
| B3 | 33.8 | 3.44 | 7,462 | 598 |
| B4 | 33.8 | 3.53 | 5,266 | 462 |
| C1 | 33 | 3.21 | 8,536 | 778 |
| C2 | 33.5 | 3.43 | 6,278 | 522 |
| D1 | 33 | 3.71 | 3,392 | 352 |
| D2 | 34 | 3.24 | 6,416 | 400 |
| D3 | 33.5 | 3.92 | 2,972 | 281 |
| D4 | 33.15 | 9.41 | 1,990 | 234 |
| E1 | 33.8 | 5.09 | 3,456 | 258 |
| E2 | 33 | 4.59 | 3,182 | 322 |
| E3 | 34 | 3.71 | 1,848 | 112 |
| F1 | 33.8 | 4.23 | 4,546 | 308 |
| F2 | 33 | 5.07 | 3,682 | 312 |
| X | 33.8 | 3.64 | 3,098 | 316 |
| MT | 155 | 54 | 440 | 6 |
| Total |  |  | 99,494 | 8,355 |

Table S10. Groups of IRs found by RepeatMasker in Fca-6.2: number of found discrete elements, length they occupy (in Mbp) and content (%) relative to the whole cat genome length.

| **Group of REs** | **Number of elements detected** | **Range of elements number in each chromosome** | **Length occupied, Mbp** | **Percentage of whole genome sequence** | **Percentage of whole genome sequence occupied by REs in**  *(from (1))* | | |
| --- | --- | --- | --- | --- | --- | --- | --- |
|  |  |  |  |  | **Dog** | **Mouse** | **Human** |
| SINEs | 1,490,125 | 28,921 – 142,645 | 262.2 | 10.80% | 10.57% | 7.96% | 13.63% |
| LINEs | 838,507 | 14,761 – 49,607 | 420.3 | 17.30% | 18.74% | 19.54% | 21.05% |
| LINE1 | 512,575 | 8,827- 50,472 | 334.1 | 13.80% | 15.57% | 19.10% | 17.43% |
| LINE2 | 273,548 | 5,214 - 29,307 | 74.8 | 3.00% | 2.84% | 0.38% | 3.25% |
| LTR elements | 304,436 | 5,870 – 30,885 | 127.2 | 5.24% | 3.68% | 10.39% | 8.62% |
| ERVL | 88,865 | 1,428 – 9,199 | 39.7 | 1.60% | 1.19% | 1.08% | 1.61% |
| ERVL-MaLRs | 145,925 | 3,179 – 14,724 | 50.5 | 2.08% | 2.05% | 4.05% | 3.79% |
| ERV I | 49,952 | 806 – 4,955 | 28.6 | 1.18% | 0.61% | 0.76% | 2.93% |
| ERV II | 774 | 4 - 82 | 4.3 | 0.18% | 0.01% | 0.00% | 0.01% |
| DNA transposons | 309,203 | 6,284 – 29,087 | 64.8 | 2.67% | 1.98% | 0.88% | 3.01% |
| Unclassified | 6,316 | 79 - 695 | 0.76 | 0.03% |  |  |  |
| Total IRs |  |  | 875 | 36.00% | 35.15% | 39.10% | 46.46% |
| TOTAL MASKED |  |  | 1,001.12 | 41.22% |  |  |  |

Table S11. Comparison of the repeat masking by WindowMasker (WM) and RepeatMasker (RM) for Fca-6.2 chromosomes except the mitochondrial one.

| Tool | Total length of the masked regions (Mbp) | Range of masked regions length across chromosomes (Mbp) | Relative length of the masked regions to genome sequence | Range of masked regions relative length across chromosomes |
| --- | --- | --- | --- | --- |
| **RM** | 1,001.12 | 16.35 – 97.32 | 41.22% | 35.99 – 52.13% |
| **WM** | 776.28 | 11.09 – 78.81 | 31.96% | 25.77 – 39.70% |

Table S12. Number of TRs detected in Fca-6.2 assembly by TRF with subsequent filtering.

| **Assembly scaffolds** | **All TRs** | **STRs** | **CTRs** | **Other TRs** |
| --- | --- | --- | --- | --- |
| Placed to chromosomes | 862,209 | 721,237 | 3,245 | 137,727 |
| Unplaced | 5,630 | 2,690 | 698 | 2,542 |

Table S13. Families of found CTRs, including previously described (items 1-3), and newly discovered (items 4-28) families.

| **Item** | **Family** | **Arrays on chromosomes** | **Arrays in ChrUn** |
| --- | --- | --- | --- |
| 1 | SL | 1,708 | 32 |
| 2 | ML5 | 555 | 36 |
| 3 | TR-483A-FC | 44 | 254 |
| 4 | TR-10A-FC | 331 | 0 |
| 5 | TR-84A-FC | 276 | 0 |
| 6 | TR-25B-FC | 53 | 0 |
| 7 | TR-113A-FC | 34 | 10 |
| 8 | TR-22A-FC | 32 | 0 |
| 9 | TR-41A-FC | 30 | 0 |
| 10 | TR-37A-FC | 29 | 0 |
| 11 | TR-25A-FC | 28 | 0 |
| 12 | TR-24A-FC | 14 | 0 |
| 13 | TR-25C-FC | 14 | 0 |
| 14 | TR-241A-FC | 11 | 0 |
| 15 | TR-15A-FC | 11 | 0 |
| 16 | TR-12A-FC | 11 | 0 |
| 17 | TR-19A-FC | 10 | 51 |
| 18 | TR-15B-FC | 10 | 0 |
| 19 | TR-30A-FC | 8 | 0 |
| 20 | TR-15C-FC | 8 | 0 |
| 21 | TR-38A-FC | 8 | 0 |
| 22 | TR-33A-FC | 8 | 233 |
| 23 | TR-15D-FC | 6 | 0 |
| 24 | TR-56A-FC | 6 | 0 |
| 25 | TR-31A-FC | 0 | 14 |
| 26 | TR-31B-FC | 0 | 28 |
| 27 | TR-68A-FC | 0 | 17 |
| 28 | TR-26A-FC | 0 | 8 |

Table S14 Absolute number (*x*10^3^*) and relative content (%) of discrete REs detected by different tools (in bold) and comparison of how they overlap to each other. Last column shows # and % of those unique REs, which were found by one of these tools and did not overlap with others. Note that different datasets include different combinations of REs groups: RM (-nolow) and WM datasets include IRs and satellite CTRs, RM (-noint) and Dustmasker both contain only STRs and low-complexity regions, while “TRF-2000” dataset is thought to contain CTRs.

| *Tool used for REs’ finding* | *Overlapping with datasets obtained by other tools* | | | | | *Unique REs* |
| --- | --- | --- | --- | --- | --- | --- |
|  | RepeatMasker – nolow | RepeatMasker – noint | WindowMasker | “TRF-2000  Workflow” | Dustmasker |  |
| RM –nolow | **100%** | 0.08% | 23.87% | 0.02% | 0.25% | 11.33% |
|  | **3,579.8** | 2.983 | 854.36 | 0.607 | 9.045 | 405.735 |
| RM –noint | 49.73% | **100%** | 85.20% | 0.03% | 27.80% | 1.36% |
|  | 997.36 | **2005.36** | 1,708.6 | 0.677 | 557.4 | 27.327 |
| WM | 34.48% | 0.42% | **100%** | 0.06% | 0.89% | 36.89% |
|  | 4,569.9 | 55.03 | **13,255.0** | 8.173 | 117 | 4,889.642 |
| “TRF-2000  Workflow” | 2.15% | 5.73% | 2.36% | **100%** | 7.99% | 29.99% |
|  | 0.062 | 0.165 | 0.068 | **2.878** | 0.23 | 0.863 |
| Dustmasker | 36.01% | 4.47% | 81.06% | 0.03% | **100%** | 6.59% |
|  | 1,669.73 | 207.421 | 3,758.311 | 1.447 | **4,636.62** | 305.529 |

Table S15. TRs detected by RepeatMasker on Fca-6.2.

| Type of TRs | Number of detected discrete elements in the genome | Range of elements number across chromosomes | Total length occupied in the genome (kbp) | Range of occupied lengths across chromosomes (kbp) | Relative length to genome sequence | Range of relative lengths across chromosomes |
| --- | --- | --- | --- | --- | --- | --- |
| CTRs | 287 | 1 – 39 | 365.289 | 0.207 – 40.209 | 0.015% | 0.00 – 0.06% |
| STRs | 1 483 118 | 24 548 – 135 618 | 70300 | 1200 – 7000 | 2.89% | 2.73 – 3.07% |

Table S16. STRs and counts

| **Type of STR** | **Count in Assembly** | **#Primers Designed** | **# Primers Mapped to Unique Locus** |
| --- | --- | --- | --- |
| PolyN | 6,609.016 | NA | NA |
| 2-mer | 700.473 | 40.420 | 39.398 |
| 3-mer | 28.728 | 5.188 | 5.042 |
| 4-mer | 73.813 | 6.411 | 6.254 |
| 5-mer | 16.261 | 1.322 | 1.288 |
| 6-mer | 3.448 | 353 | 345 |
| 7-mer | 244 | 16 | 16 |
| Total STR | 822.967 | 53.710 | 52.343 |

Table S17. Summary of ECEs in Fca-6.2 genome assembly

| **Chr** | **# ECEs** | **Total, bp** | **% of chr** |
| --- | --- | --- | --- |
| A1 | 69,369 | 6,709,971 | 2.80 |
| A2 | 57,775 | 5,309,955 | 3.14 |
| A3 | 45,456 | 4,250,851 | 2.98 |
| B1 | 52,481 | 4,795,627 | 2.34 |
| B2 | 41,795 | 3,830,365 | 2.48 |
| B3 | 47,286 | 4,646,282 | 3.13 |
| B4 | 45,023 | 4,077,498 | 2.83 |
| C1 | 81,273 | 8,088,931 | 3.65 |
| C2 | 44,557 | 4,143,226 | 2.63 |
| D1 | 35,267 | 3,243,109 | 2.77 |
| D2 | 28,247 | 2,751,194 | 3.06 |
| D3 | 25,842 | 2,427,503 | 2.54 |
| D4 | 31,838 | 3,051,314 | 3.18 |
| E1 | 27,070 | 2,564,827 | 4.07 |
| E2 | 23,108 | 2,483,270 | 3.88 |
| E3 | 16,194 | 1,434,914 | 3.34 |
| F1 | 23,233 | 2,061,812 | 3.00 |
| F2 | 20,090 | 1,912,468 | 2.31 |
| X | 23,754 | 1,873,010 | 1.48 |
| MT | 7 | 259 | 1.52 |
| Unlocalized | 3,557 | 342,627 | 2.23 |
| Unplaced | 140 | 9,201 | 0.08 |
| **Total** | **743,362** | **70,008,214** |  |

Table S18. Conserved sequence blocks (CSB) derived from reciprocal best matches with a number of reference genomes. SD stands for standard deviation.

| **Reference Genome Set** | **Length (in BP)** | | | **# CSB** |
| --- | --- | --- | --- | --- |
|  | **Mean** | **SD** | **Max** |  |
| A: dog and cow | 1,140 | 1,021 | 17,763 | 728,023 |
| B: dog, cow, human, chimpanzee and macaque | 967 | 819 | 15,317 | 572,097 |
| C: dog, cow, human, chimpanzee, macaque, mouse and rat | 722 | 629 | 11,183 | 252,583 |

Table S19a. Results of aligning viral sequences to Fca-6.2 assembly.

| **Virus** | **Total length of alignments (in kb)** | **Number of alignments** |
| --- | --- | --- |
| enFeLV | 140.38 | 24 |
| FeLV | 11.38 | 1 |
| FERV | 1,535.11 | 125 |
| FeSV | 17.35 | 4 |
| RD114 | 375.85 | 12 |
| Syncytin | 517.47 | 44 |
| Other sequences | 1,034.65 | 263 |
| Total | 3,632.19 | 473 |

Table S19B. Results of aligning viral pol gene sequences to Fca-6.2 assembly.

| **Virus host species** | **Total length of alignments (in kb)** | **Number of alignments** |
| --- | --- | --- |
| Baboon | 24 | 9 |
| Cat | 13 | 39 |
| Cougar | 2 | 3 |
| Dog | 59 | 163 |
| Human | 4 | 23 |
| Mouse | 27 | 16 |
| Pig | 19 | 40 |
| Sheep | 22 | 34 |

TABLE S20. Methylated cytosine residues in domestic cat white blood cells

| **Chr** | **# C** | **# G** | **# mC** | **% mC** |
| --- | --- | --- | --- | --- |
| chrA1 | 46,531,955 | 46,529,589 | 9,100,254 | 9.78% |
| chrA2 | 34,439,295 | 34,469,037 | 7,470,698 | 10.84% |
| chrA3 | 29,547,783 | 29,576,180 | 6,492,200 | 10.98% |
| chrB1 | 38,869,549 | 38,919,756 | 7,383,498 | 9.49% |
| chrB2 | 29,943,446 | 29,948,701 | 5,964,676 | 9.96% |
| chrB3 | 29,900,930 | 30,026,043 | 6,278,723 | 10.48% |
| chrB4 | 28,737,216 | 28,742,747 | 6,029,391 | 10.49% |
| chrC1 | 44,586,757 | 44,627,440 | 9,136,541 | 10.24% |
| chrC2 | 30,392,995 | 30,310,438 | 5,846,934 | 9.63% |
| chrD1 | 23,814,350 | 23,925,732 | 5,036,850 | 10.55% |
| chrD2 | 18,530,780 | 18,513,414 | 4,302,984 | 11.62% |
| chrD3 | 19,742,549 | 19,722,174 | 4,658,406 | 11.80% |
| chrD4 | 19,538,235 | 19,496,787 | 4,355,976 | 11.16% |
| chrE1 | 13,855,578 | 13,826,829 | 3,623,471 | 13.09% |
| chrE2 | 13,743,239 | 13,755,214 | 3,403,158 | 12.38% |
| chrE3 | 9,515,684 | 9,474,206 | 2,680,680 | 14.12% |
| chrF1 | 14,425,295 | 14,417,044 | 3,363,025 | 11.66% |
| chrF2 | 16,610,093 | 16,568,045 | 3,472,203 | 10.47% |
| chrMT | 4,454 | 2,406 | 6,272 | 91.43% |
| chrX | 24,259,474 | 24,299,103 | 3,837,311 | 7.90% |
| Total | 486,989,657 | 487,150,885 | 102,443,251 | 10.52% |

Table S21 Statistics on species which miRNA sequences for miRBase database formed the alignments the putative cat miRNA regions were derived from.

| **Species** | **# miRNAs** |
| --- | --- |
| Anolis carolinensis | 32 |
| Artibeus jamaicensis | 20 |
| Ateles geoffroyi | 37 |
| Bos taurus | 258 |
| Canis familiaris | 265 |
| Cricetulus griseus | 105 |
| Cyprinus carpio | 7 |
| Danio rerio | 12 |
| Equus caballus | 246 |
| Fugu rubripes | 10 |
| Gallus gallus | 47 |
| Gorilla gorilla | 150 |
| Hippoglossus hippoglossus | 1 |
| Homo sapiens | 270 |
| Ictalurus punctatus | 15 |
| Lagothrix lagotricha | 40 |
| Lemur catta | 14 |
| Macaca mulatta | 229 |
| Macaca nemestrina | 59 |
| Monodelphis domestica | 82 |
| Mus musculus | 167 |
| Ornithorhynchus anatinus | 38 |
| Oryzias latipes | 4 |
| Ovis aries | 61 |
| Pan paniscus | 73 |
| Pan troglodytes | 240 |
| Paralichthys olivaceus | 4 |
| Pongo pygmaeus | 224 |
| Rattus norvegicus | 151 |
| Saguinus labiatus | 32 |
| Sarcophilus harrisii | 7 |
| Sus scrofa | 200 |
| Taeniopygia guttata | 52 |
| Tetraodon nigroviridis | 12 |
| Xenopus laevis | 1 |
| Xenopus tropicalis | 17 |
| Total: | 3,182 |

Table S2**2** Summary of 1-Kbps windows, copy number distribution in control regions and gain/loss cutoffs**.**

| **Sequencing** |  |
| --- | --- |
| Sequencing technology | Illumina |
| # Reads | 1,485,609,004 |
| Coverage | 21.8X |
|  |  |
| **1-Kbps windows** |  |
| # Total windows | 1,122,501 |
| # Control windows | 993,102 |
| # Non control windows | 129,399 |
|  |  |
| **Gain/loss cutoffs** |  |
| Mean copy number in control regions | 2.00 |
| StDev copy number in control regions | 0.24 |
| (# windows excluded*) | 9,932 |
| Gain cutoff | 2.71 |
| Loss cutoff | 1.29 |

*1-Kbps windows exceeding the 1% highest copy number value.

**Table S23** Autosomal duplications detected using the depth of coverage. All bps are after excluding the size of the gaps.

|  | **M1** |
| --- | --- |
| # Total bps | 9,340,141 |
| % genome | 0.4 |

SUPPLEMENTAL FIGURE

Figure S1. Architecture of GARfield browser.


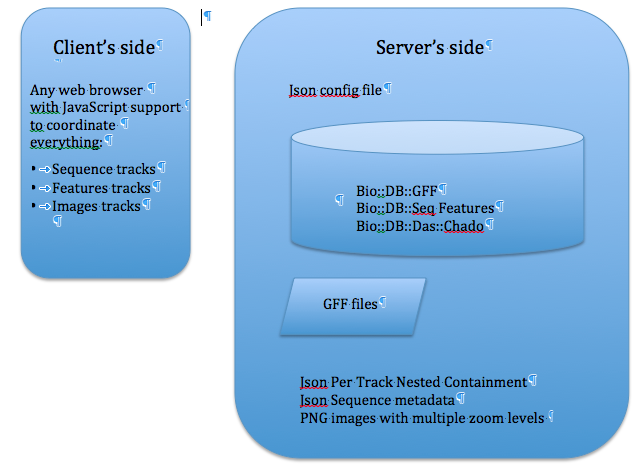


Figure S2 Fractions of SNVs annotated per cat chromosome


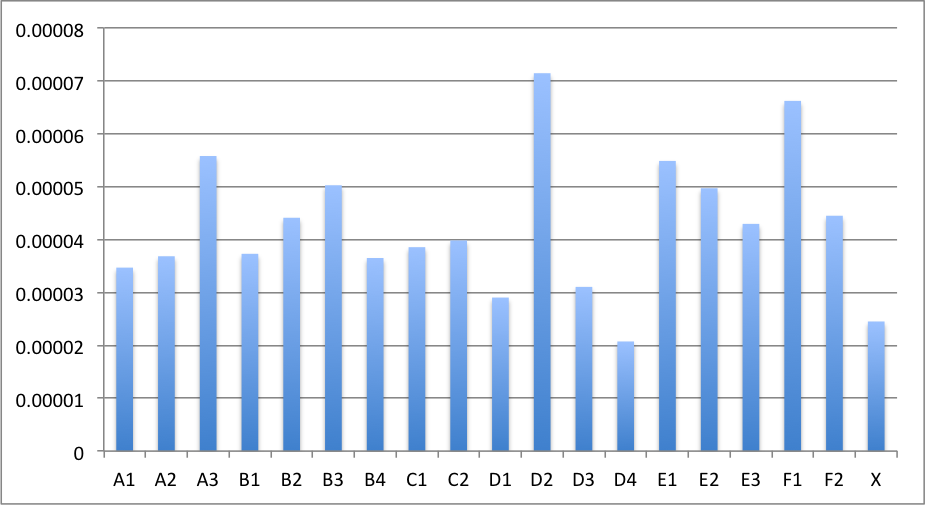


Figure S3 Fractions of indels annotated per cat chromosome.


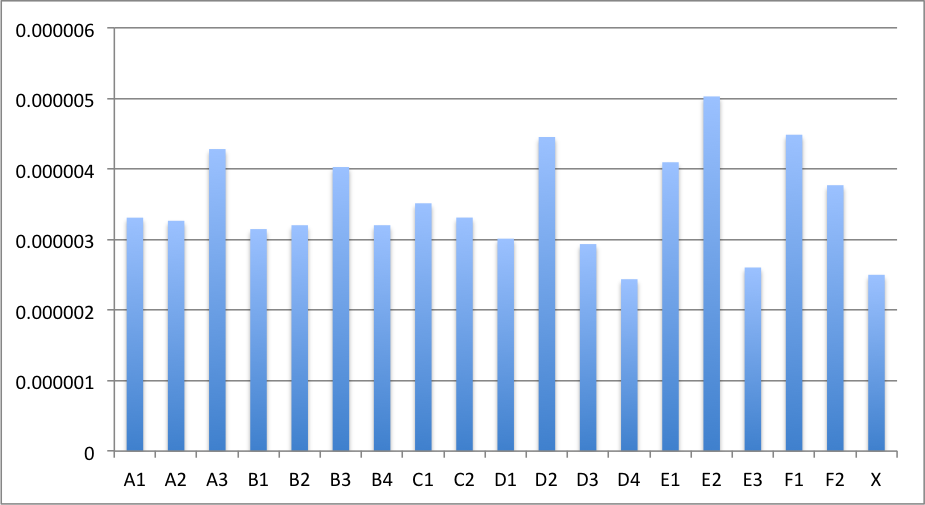


Figure S4. Absolute number (axis y) of different families of REs (axis x) found by RepeatMasker in the whole genome of domestic cat.


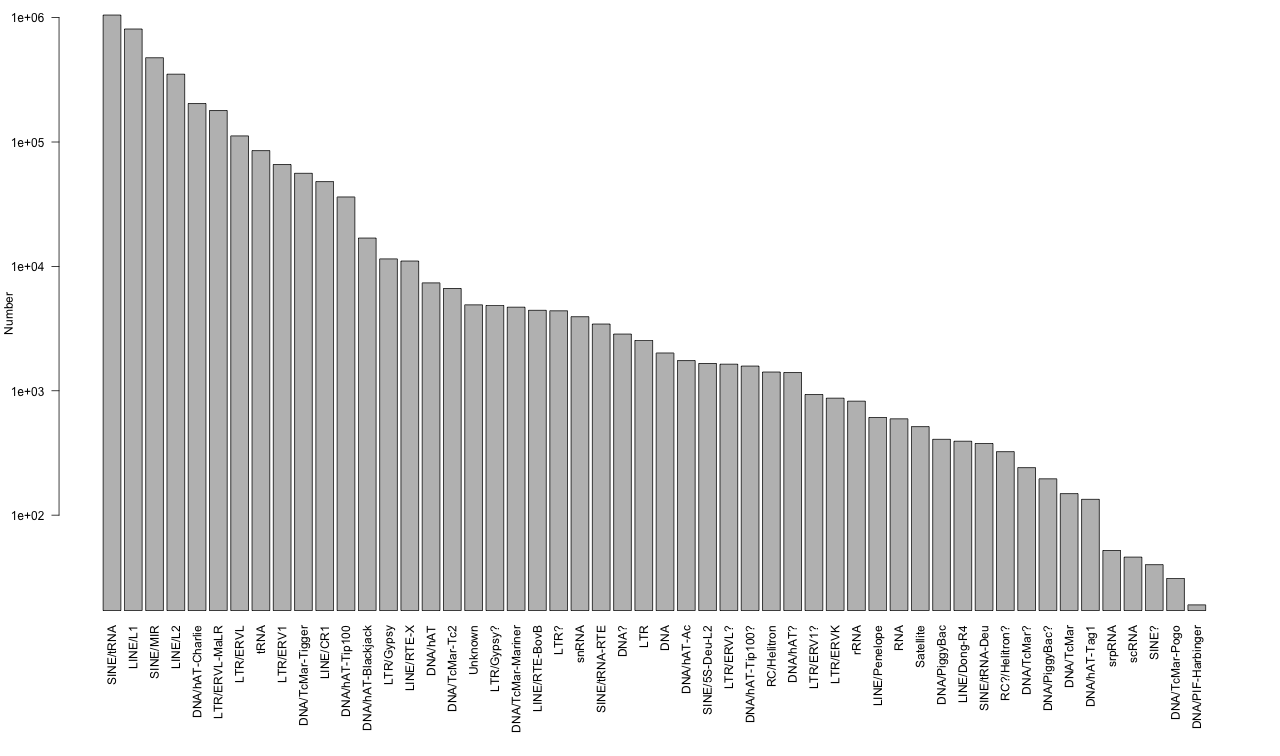


Figure S5. Relative content of RE classes across chromosomes in domestic cat.


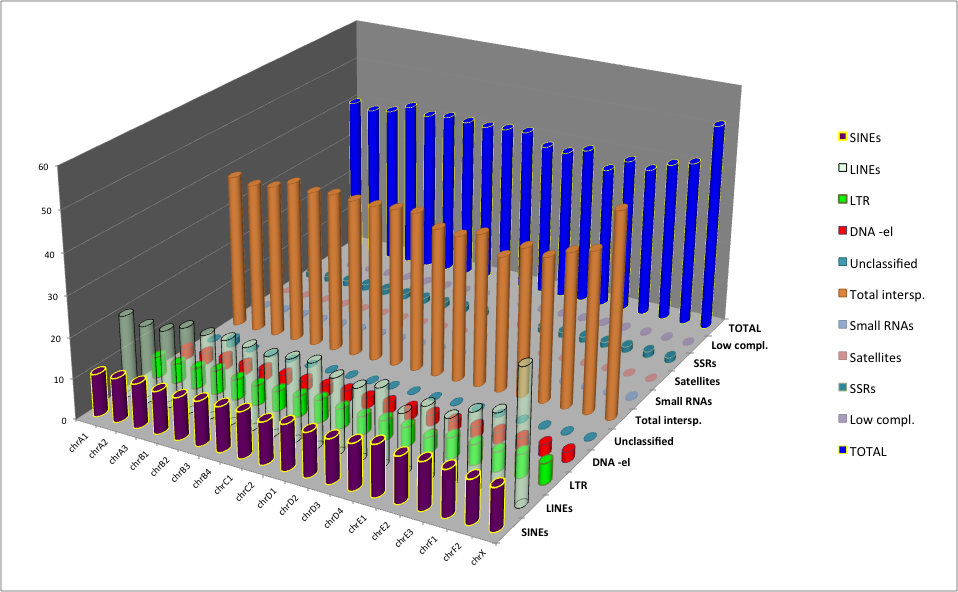


Figure S6. Comparison of REs detected by RM and WM. “Combined” corresponds to REs derived by combining of RM and WM repeats.


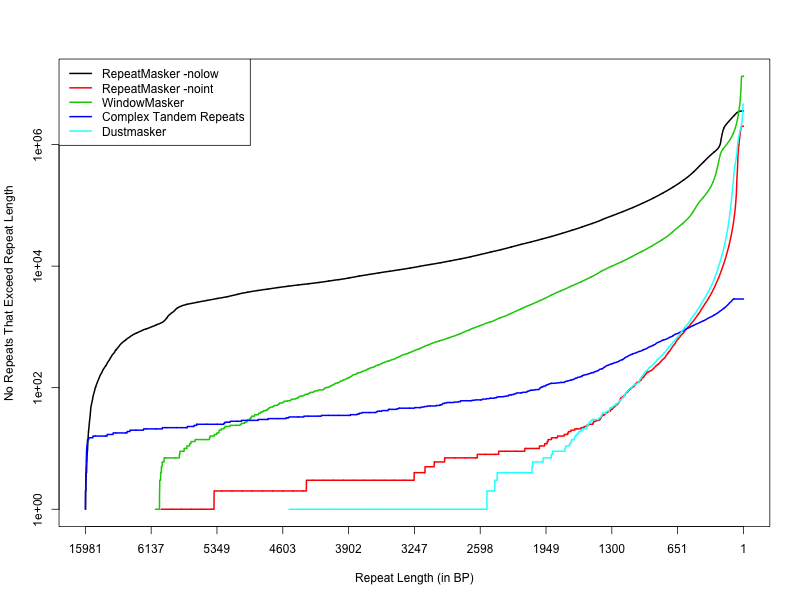


Figure S7. Nomenclature of complex tandem repeats.


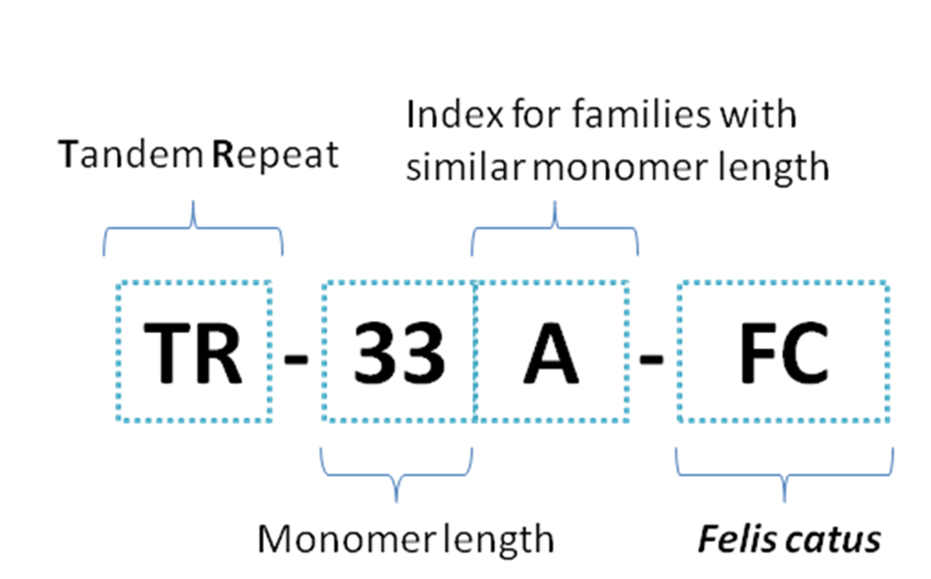


Figure S8. **A**. The distribution of complex tandem repeats from the reference assembly according to GC-content, monomer length, and monomer similarity in array. Each sphere represents one array. Spheres are colored according to given legend. **B**. Shown only 14 largest families.


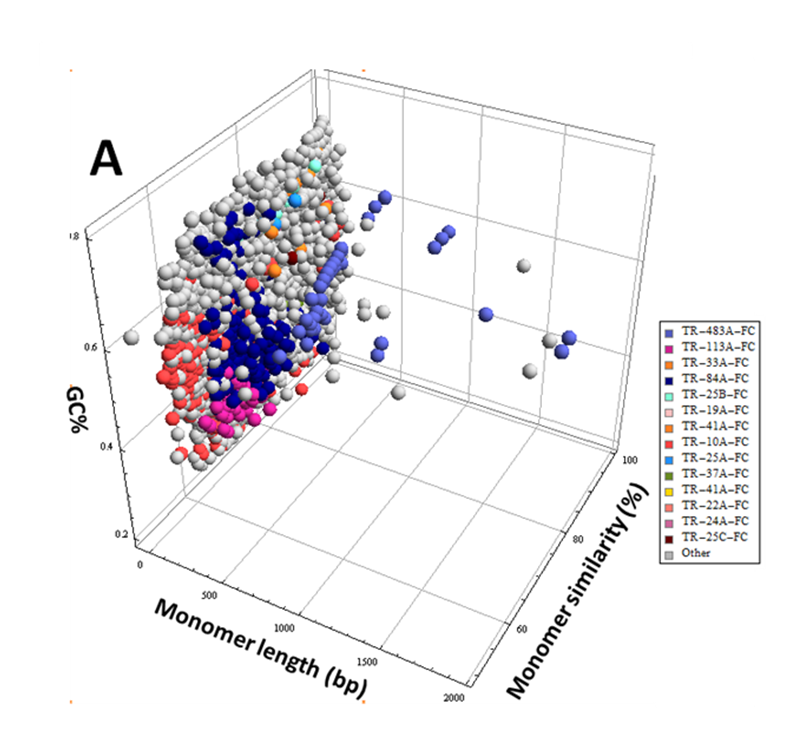


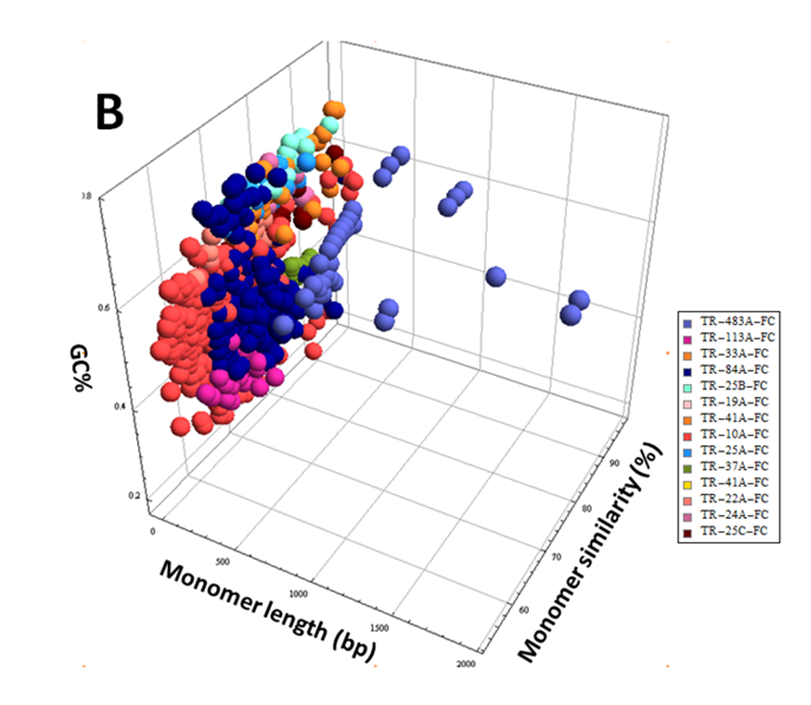


Figure S9. Position of all CTRs on the Fca-6.2. Centromeric gaps are marked with asterisk. Band intensity shown according to sequence length of localized repeats.


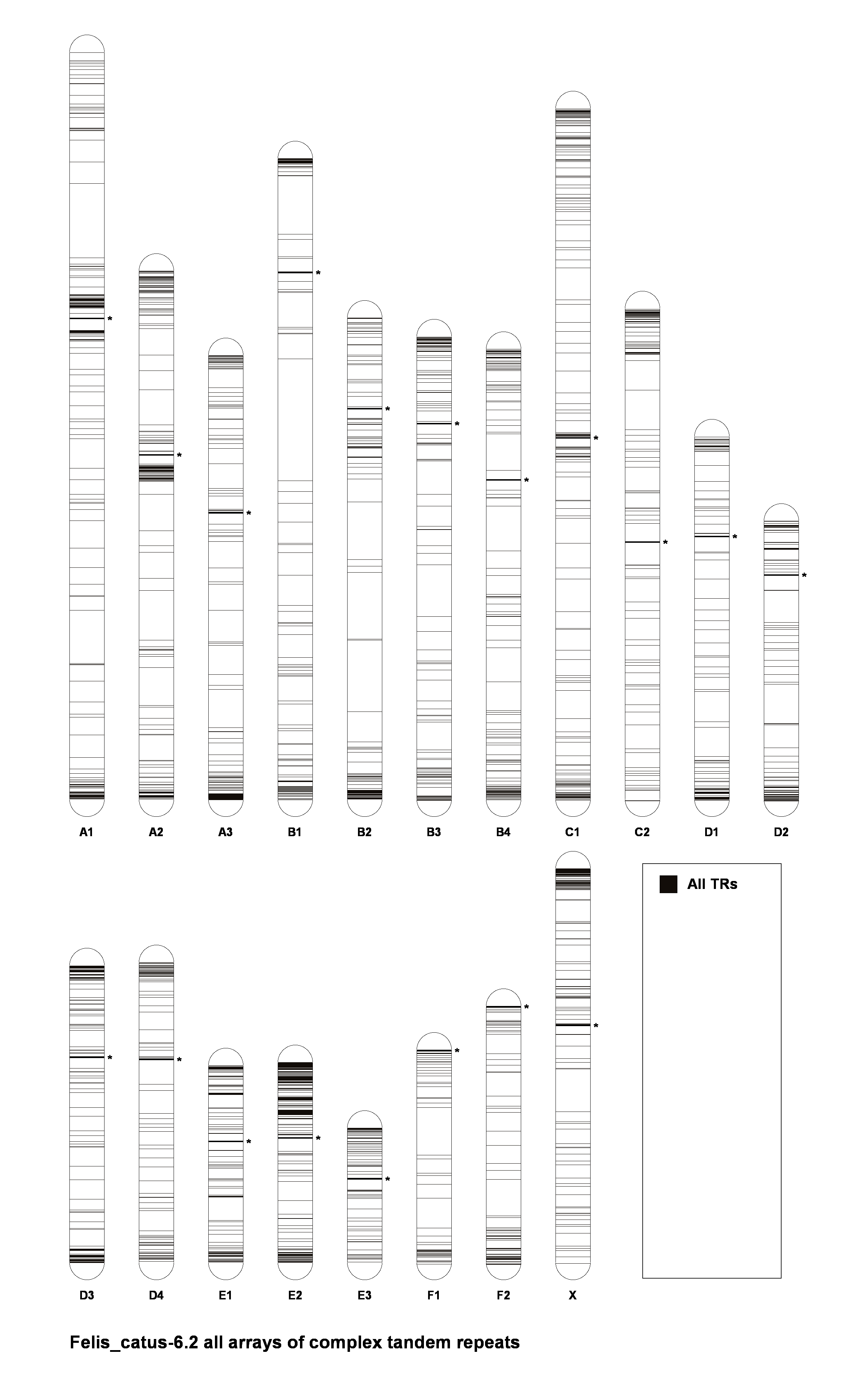


Figure S10. Position of single locus CTRs on the Fca-6.2.


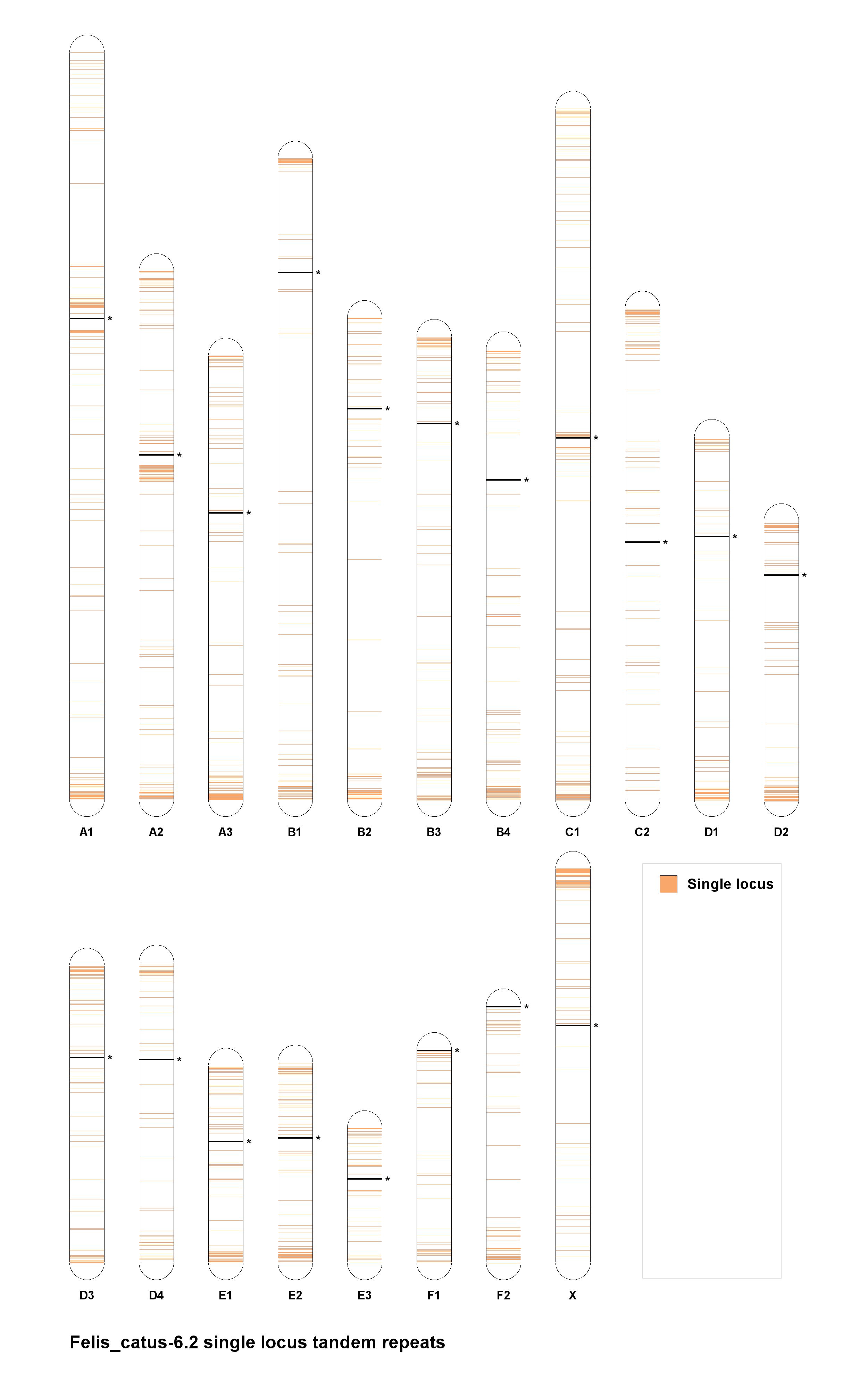


Figure S11. Position of ML5 CTRs (less than 6 loci) on the Fca-6.2.


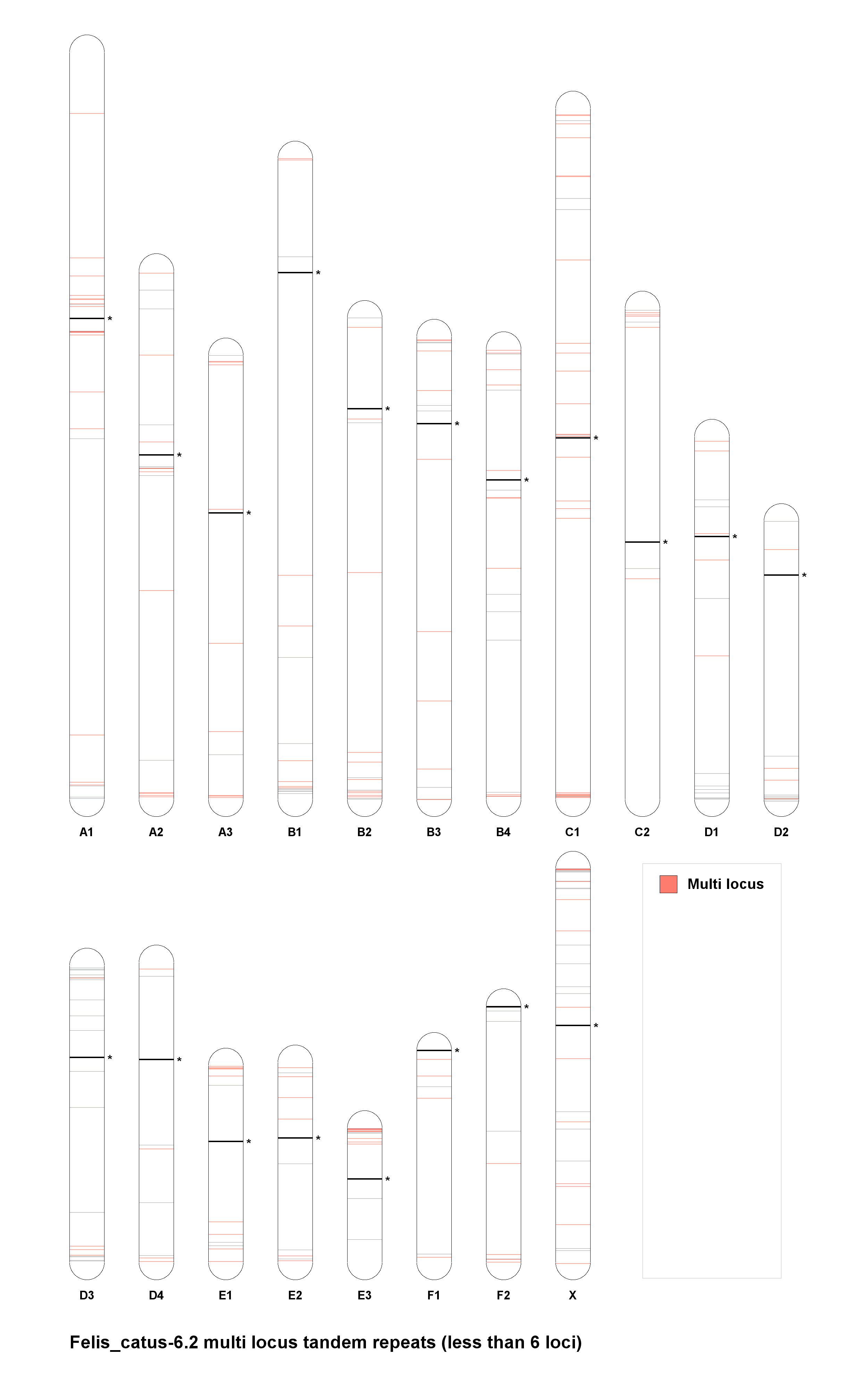


Figure S12. Position of multi locus CTRs (more than 11 loci) on the Fca-6.2.


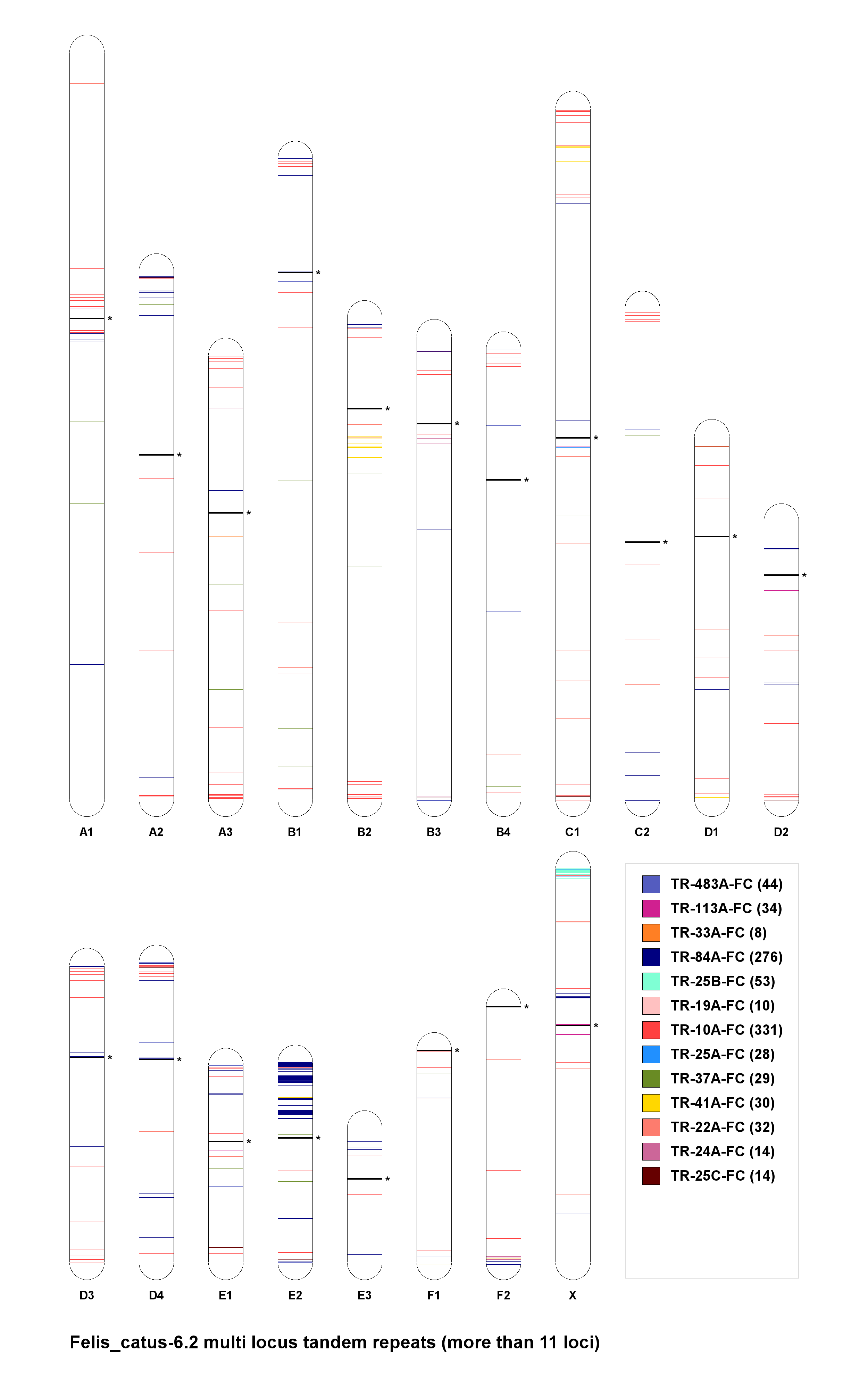


Figure S13. Position of FA-SAT elements on the Fca-6.2.


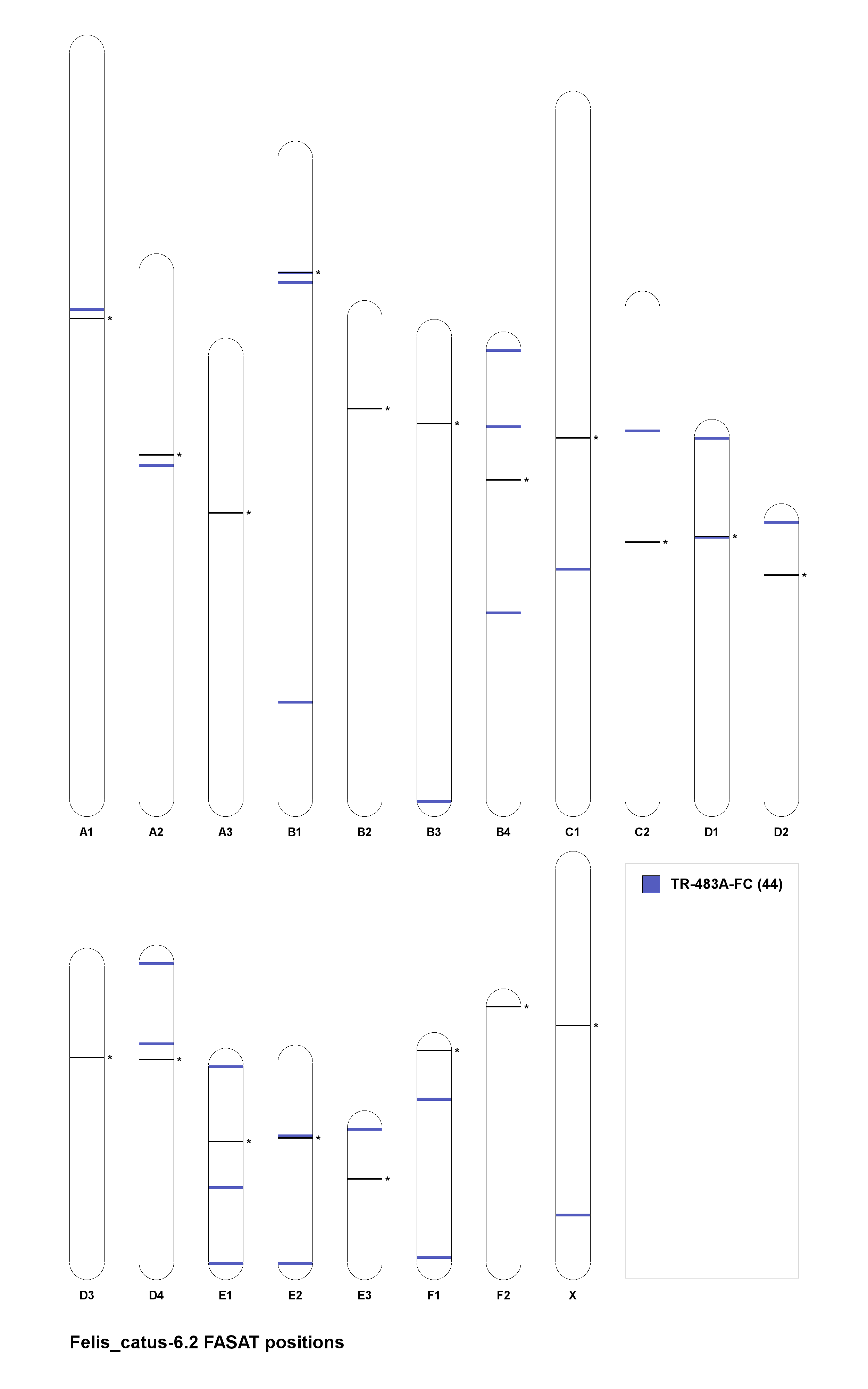


Figure S14. Proportion of *numt* fragments assigned to the domestic cat chromosomes. (A) Data from the previous 1.9x coverage of the *F. catus* genome (1,60). (B) Data from the *F. catus* genome Fca-6.2. 298,320 bp of *numts* covering 99% of the mtDNA genome from the previous 1.9x coverage of the *F. catus* genome, which likely contained redundant sequences not assigned to chromosomes(1,60).

**A**


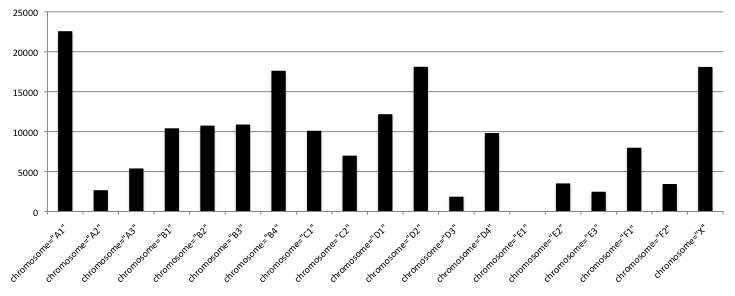


**B**

**Figure S15.** Cumulative distribution of additional masking achieved by masking over-represented kmers in Fca 6.2 (FelCat5 in UCSC)

**
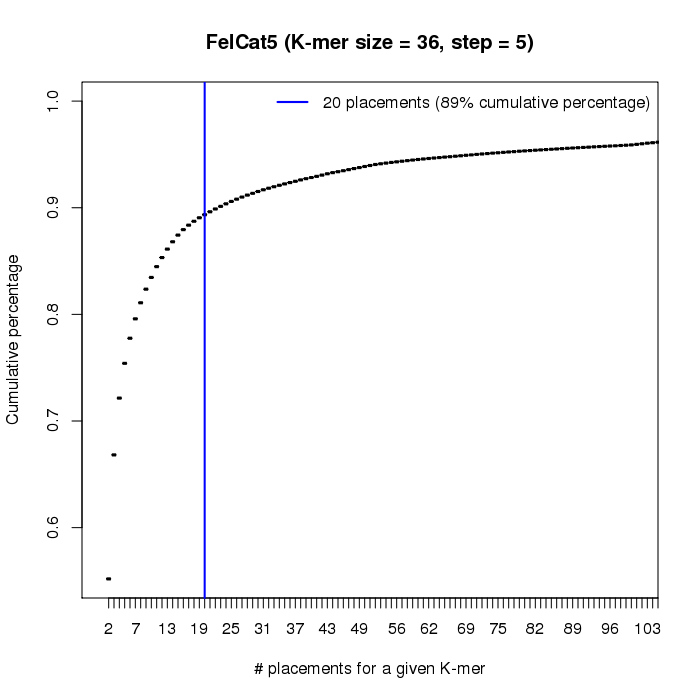
**

**Figure S16.** Distribution of 1-Kbps copy number values in control and non-control regions**.** The number of windows in each distribution is indicated.


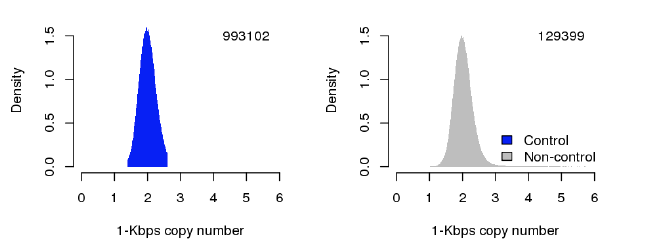


**Figure S17.** CNV map on domestic cat autosomes based on depth of coverage.


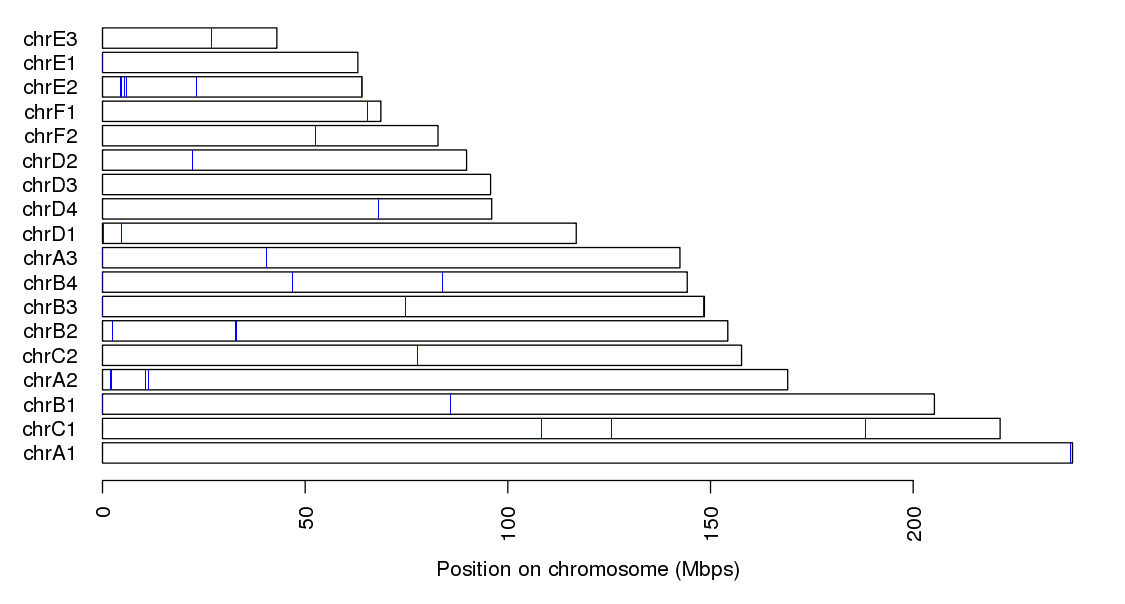


**Figure S18.** Phylogenetic tree of the cat genome regions similar to retroviral *pol* genes. Tip labels correspond to the original viral sequence groups that formed alignments with the cat genome. Groups related to human are in blue color, to pig in green color, and to dog in red color. The tree branches were supported by bootstrap (> 50%).


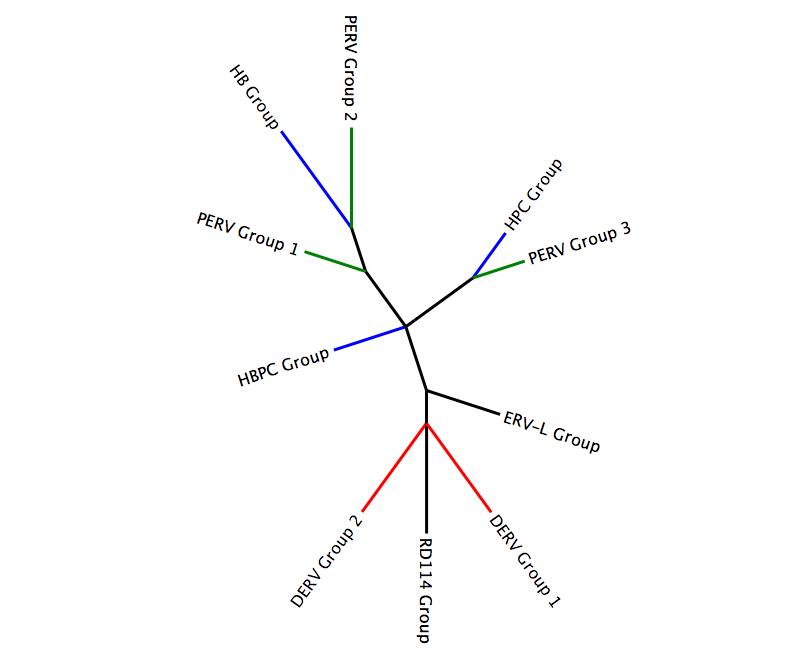

Supplement: Additional file 1 — Supplementary materials. [file 2047-217X-3-13-S1.docx]
